# AAV‐Mediated nuclear localized PGC1α4 delivery in muscle ameliorates sarcopenia and aging‐associated metabolic dysfunctions

**DOI:** 10.1111/acel.13961

**Published:** 2023-08-16

**Authors:** Mingwei Guo, Jun Zhang, Ying Ma, Zhenzhong Zhu, Hui Zuo, Jing Yao, Xia Wu, Dongmei Wang, Jian Yu, Meiyao Meng, Caizhi Liu, Yi Zhang, Jiangrong Chen, Jian Lu, Shuzhe Ding, Cheng Hu, Xinran Ma, Lingyan Xu

**Affiliations:** ^1^ Shanghai Key Laboratory of Regulatory Biology Institute of Biomedical Sciences and School of Life Sciences, East China Normal University Shanghai China; ^2^ Department of Orthopedics Sixth People's Hospital Affiliated to Shanghai Jiao Tong University School of Medicine Shanghai China; ^3^ Department of Endocrinology and Metabolism Fengxian Central Hospital Affiliated to Southern Medical University Shanghai China; ^4^ Shanghai Diabetes Institute, Shanghai Key Laboratory of Diabetes Mellitus Shanghai Clinical Center for Diabetes, Shanghai Jiao Tong University Affiliated Sixth People's Hospital Shanghai China; ^5^ Key Laboratory of Adolescent Health Assessment and Exercise Intervention of Ministry of Education, College of Physical Education and Health East China Normal University Shanghai China; ^6^ Shanghai Frontiers Science Center of Genome Editing and Cell Therapy, Shanghai Key Laboratory of Regulatory Biology and School of Life Sciences East China Normal University Shanghai China; ^7^ Chongqing Key Laboratory of Precision Optics Chongqing Institute of East China Normal University Chongqing China

**Keywords:** aging, metabolic dysfunctions, nuclear localization, PGC1α4, sarcopenia

## Abstract

Sarcopenia is characterized of muscle mass loss and functional decline in elder individuals which severely affects human physical activity, metabolic homeostasis, and life quality. Physical exercise is considered effective in combating muscle atrophy and sarcopenia, yet it is not feasible to elders with limited mobility. PGC‐1α4, a short isoform of PGC‐1α, is strongly induced in muscle under resistance training, and promotes muscle hypertrophy. In the present study, we showed that the transcriptional levels and nuclear localization of PGC1α4 was reduced during aging, accompanied with muscle dystrophic morphology, and gene programs. We thus designed NLS‐PGC1α4 and ectopically express it in myotubes to enhance PGC1α4 levels and maintain its location in nucleus. Indeed, NLS‐PGC1α4 overexpression increased muscle sizes in myotubes. In addition, by utilizing AAV‐NLS‐PGC1α4 delivery into gastrocnemius muscle, we found that it could improve sarcopenia with grip strength, muscle weights, fiber size and molecular phenotypes, and alleviate age‐associated adiposity, insulin resistance and hepatic steatosis, accompanied with altered gene signatures. Mechanistically, we demonstrated that NLS‐PGC‐1α4 improved insulin signaling and enhanced glucose uptake in skeletal muscle. Besides, via RNA‐seq analysis, we identified myokines IGF1 and METRNL as potential targets of NLS‐PGC‐1α4 that possibly mediate the improvement of muscle and adipose tissue functionality and systemic energy metabolism in aged mice. Moreover, we found a negative correlation between PGC1α4 and age in human skeletal muscle. Together, our results revealed that NLS‐PGC1α4 overexpression improves muscle physiology and systematic energy homeostasis during aging and suggested it as a potent therapeutic strategy against sarcopenia and aging‐associated metabolic diseases.

## Graphical abstract

Illustration figure highlighting that AAV‐mediated MCK driven nuclear localized PGC1a4 delivery in muscle ameliorates sarcopenia and aging‐associated metabolic dysfunctions.

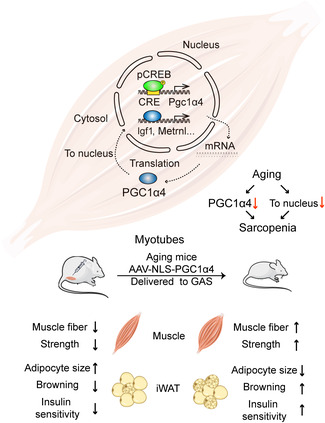

## 1 INTRODUCTION

Sarcopenia is a progressive skeletal muscle atrophy, which is characterized of profound muscle mass loss, and functional decline during aging. Sarcopenia may lead to increased incidents of falls, bone fractures, frailty and mortality, thus severely affects physical activity and life quality in elder population (Cruz‐Jentoft & Sayer, 2019; Larsson et al., 2019). Although nutrient supplementation and physical activity are considered to be effective in countering muscle atrophy, these interventions may be difficult to implement in elders with special diet habits, malabsorption, or limited mobility (Dasarathy & Merli, 2016). Thus, novel strategies and compounds aiming to combat muscle atrophy is urgently needed.

Resistance exercise ameliorates muscle atrophy via different regulatory pathways, the mechanisms of which have been extensively studied (Vainshtein & Sandri, 2020). Fortunately, with the advancement of gene therapy, it is now possible to utilize gene therapy to mimic or augment the beneficial effects of exercises to combat muscle atrophy. For example, in vivo administration of specific myostatin antagonist or CRISPR/Cas9 mediated disruption of myostatin gene, a well‐established cytokine that activated in muscle atrophy, has been shown to effectively alleviate muscle wasting (Li et al., 2020; Wei et al., 2016). Besides, AAV mediated Smad7 gene delivery prevents cancer cachexia‐associated muscle wasting in mice by abolishing SMAD2/3 signaling downstream of ActRIIB and inhibiting atrophy‐related genes expression (Winbanks et al., 2016). Meanwhile, inhibition of non‐coding RNA including miR29b or lncRNA MAAT also ameliorated multiple types of muscle atrophy (Li et al., 2020; Li et al., 2021). However, the potential target against aging‐associated sarcopenia is still lacking.

Peroxisome proliferator‐activated receptor γ (PPARγ) coactivator‐1α (PGC‐1α) is one of the most studied transcriptional cofactors in the metabolic field for its vital roles in mitochondrial biogenesis and energy homeostasis (Martinez‐Redondo et al., 2015). Recent studies have shown the existence of different isoforms of PGC‐1α in muscle that feature distinct transcript variants and protein structures, which are produced by usage of different promoters or by alternative splicing in response to various physiological stimuli (Chinsomboon et al., 2009; Lin et al., 2005; Ruas et al., 2012; Wen et al., 2014; Yoshioka et al., 2009; Zhang et al., 2009). The use of proximal promoter located immediate to the 5′ of exon 1 produces PGC‐1α1, while the use of alternative promoter located approximately 13 kb upstream gives rise to PGC‐1α2, 3, and 4. Of note, compared to PGC‐1α1, which is mainly induced in response to endurance exercise, PGC‐1α4, a short isoform of PGC‐1α, is strongly induced in muscle under resistance training and promoted muscle hypertrophy in both basal condition and in muscle atrophic scenarios, that is, hindlimb suspension and cancer‐induced cachexia, possibly via histone modifications of its target genes including Igf1 and myostatin in nucleus (Ruas et al., 2012; White et al., 2014). It would thus be worthwhile to study the localization of PGC‐1α4 in muscle under physiological and pathological conditions and decipher whether forced enhancement of PGC‐1α4 with nuclear localization in muscle could exert its beneficial functions on muscle.

In the present study, we found that PGC1α4 transcription and nucleus localization in muscles were reduced in mice with aging‐associated sarcopenia. Considering that PGC1α4 exerts its functionality as cofactors in nuclear, we hypothesized that enhancing PGC‐1α4 levels and maintaining its retention in nucleus by ectopically expressing a modified PGC1α4 tethering to a nuclear localization signal (NLS) peptide (NLS‐PGC1α4) would enhance its functionality to alleviate sarcopenia. Indeed, NLS‐PGC1α4 overexpression increased muscle fiber sizes in myotubes, as well as enhanced viability, proliferation, and differentiation in myoblasts. Moreover, we designed and successfully applied muscle‐specific promoter MCK driven AAV to mediate the delivery of NLS‐PGC1α4 into gastrocnemius (GAS) muscle and alleviated aging‐associated sarcopenia and metabolic disorders, including adiposity, insulin resistance and fatty liver in mice, at least partially via myokines IGF1 and METRNL to improve muscle and adipose tissue functionality and systemic energy metabolism in aged mice. In addition, we showed clinical relevance of PGC‐1α4 and sarcopenia. Overall, these data suggested NLS‐PGC1α4 delivery as a potent therapeutic strategy against sarcopenia and aging‐associated metabolic dysfunctions.

## 2 RESULTS

### 2.1 PGC1α4 transcription levels are decreased in muscle during aging

**FIGURE 1:**
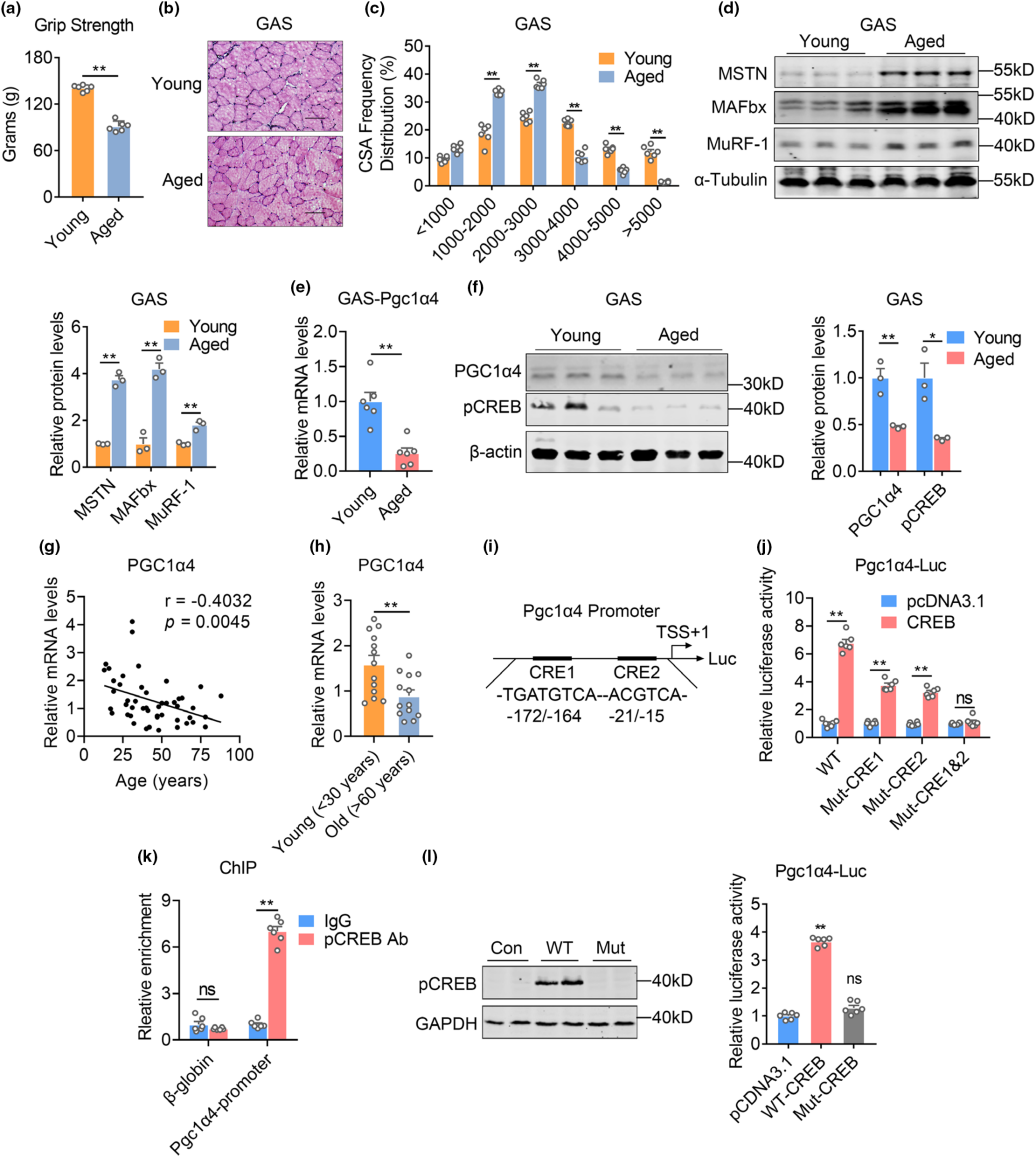
PGC1α4 transcription levels are decreased in muscle during aging. (a–d) Phenotypical and molecular analysis of muscles from young (2 months‐old) and aged mice (24 months‐old). *n* = 6 per group. (a) The grip strength; (b) H&E histological analysis; (c) CSA frequency distribution of fiber sizes; (d) Immunoblotting and quantification analysis of atrophic and inflammatory genes from GAS muscle. (e) Real time‐PCR analysis of Pgc1α4 in GAS from young (2 months‐old) and aged mice (24 months‐old). *n* = 6 per group for young and aged mice. (f) Levels of PGC1α4 and phosphorylated S133‐CREB protein in GAS from young (2 months‐old) and aged mice (24 months‐old) and density quantification of protein levels. *n* = 3 per group for young and aged mice. (g) Person analysis on the correlation between relative mRNA expression levels of Pgc1α4 and age in human vastus lateralis muscle biopsies. *n* = 48. (h) The relative mRNA expression levels of Pgc1α4 in human vastus lateralis muscle biopsies from young (<30 years) and old (>60 years). *n* = 13 per group for young and old human individuals. (i) Schematic diagram depicting CRE regions on Pgc1α4 promoter, CRE1: TGATGTCA (172 bp/−164) and CRE2: ACGTCA (−21/−15). (j) Luciferase assays showing relative luciferase activities of CREB on wildtype (WT), mutated CRE1 (Mut‐CRE1), CRE2 (Mut‐CRE2) or CRE1&2 (Mut‐CRE1&2) on Pgc1α4 promoter. *n* = 6 biological replicates. (k) in vivo ChIP assays showing S133 phosphorylated CREB occupancy on the Pgc1α4 promoter in GAS of 8‐week mice. β‐globin was served as negative control. *n* = 6 biological replicates. (l) Western blot analysis of wild type phosphorylated CREB and mutated S133A in HEK293T cell line (left panel) and luciferase assays assessing binding of WT‐CREB or Mut‐CREB on Pgc1α4 promoter (right panel). *n* = 6 biological replicates. Data are presented as mean ± SEM and *p < 0.05, **p < 0.01 compared to control group.

Compared to young mice (2‐month‐old), aged mice (24‐month‐old) showed characteristics of aging‐associated sarcopenia, which were manifested as reduced grip strength and muscle fiber sizes, as well as increased dystrophic gene expressions (Figures 1a‐d). To understand PGC1α4 expression levels and its cellular distribution in muscle under aging‐associated sarcopenia, we investigated GAS muscle from young or aged mice and found significantly decreased PGC1α4 mRNA and protein levels in aged mice (Figure 1e and Figure S1A), accompanied with reduced levels of phosphorylated cAMP responsive element binding protein (CREB), a master transcriptional regulator for skeletal muscle hypertrophy (Figure 1f). Interestingly, the expression levels of Pgc1α4 were aged‐dependent decreased in GAS muscle but were comparable in soleus muscle between young and aged mice (Figure S1A). In order to assess the clinical relevance between PGC‐1α4 and sarcopenia, we collected human muscle biopsies at different age and examined Pgc1α4 mRNA levels. The results showed a negative correlation between Pgc1α4 and age in human skeletal muscle by Pearson analysis (Figures 1g). Besides, Pgc1α4 levels declined in old individuals compared with young ones (Figures 1h), suggesting the importance of PGC1α4 as a potential target against sarcopenia in clinic.

Via in silico analysis, we identified two putative CRE binding sites on PGC1α4 promoter at ‐172 bp to ‐164 bp (CRE1) and ‐21 bp to ‐15 bp (CRE2) region (Figure 1i). Indeed, luciferase assay showed that CREB strongly induced Pgc1α4 promoter transcriptional activity, while deletion of both CRE regions on Pgc1α4 promoter fully blunted this activation (Figure 1j). Besides, chromatin immunoprecipitation (ChIP) analysis confirmed the binding of phosphorylated CREB on Pgc1α4 promoter spanning two CRE regions (CRE1 and CRE2) (Figure 1k). Moreover, as the phosphorylation of CREB is required for the activation of its downstream signaling, we found that wild type CREB dramatically activated Pgc1α4 promoter transcriptional activity, which was abrogated with a Serine133 to Alanine (Zhang et al., 2016) mutant CREB (Mut‐CREB) that lacks phosphorylation capability (Figures 1l). Thus, these data indicated the PGC1α4 levels were decreased in muscle in aged mice and old human individuals at least partially due to a decline of phosphorylated CREB in these mice for Pgc1α4 transcriptional activation.

Besides, other Pgc1α isoforms in GAS muscle, including Pgc1α1, Pgc1α2, and Pgc1α3, were all decreased in aged mice (Figure S1B). This is in consistent with previous reports that CREB serves as a master regulator for classic Pgc1α, namely Pgc1α1 (Lopez‐Lluch et al., 2008; Yoshioka et al., 2009), while Pgc‐1α2 and Pgc‐1α3 share common promoters with Pgc‐1α4 (Martinez‐Redondo et al., 2016).

### 2.2 PGC‐1α4 localization in nucleus is reduced in muscle during aging

A comparison of DNA sequences between PGC1α1 and PGC1α4 revealed the existence of NLS in C‐terminal region of PGC1α1, but not in PGC1α4. Indeed, when a GFP‐fused PGC1α4 was overexpressed in HEK293T and NIH3T3 cell lines, the GFP fluorescence showed that PGC1α4 localized mainly in cytoplasm, which was further confirmed by immunoblotting of GFP in nucleus and cytoplasm fractions (Figures S2A‐S2D).

**FIGURE 2:**
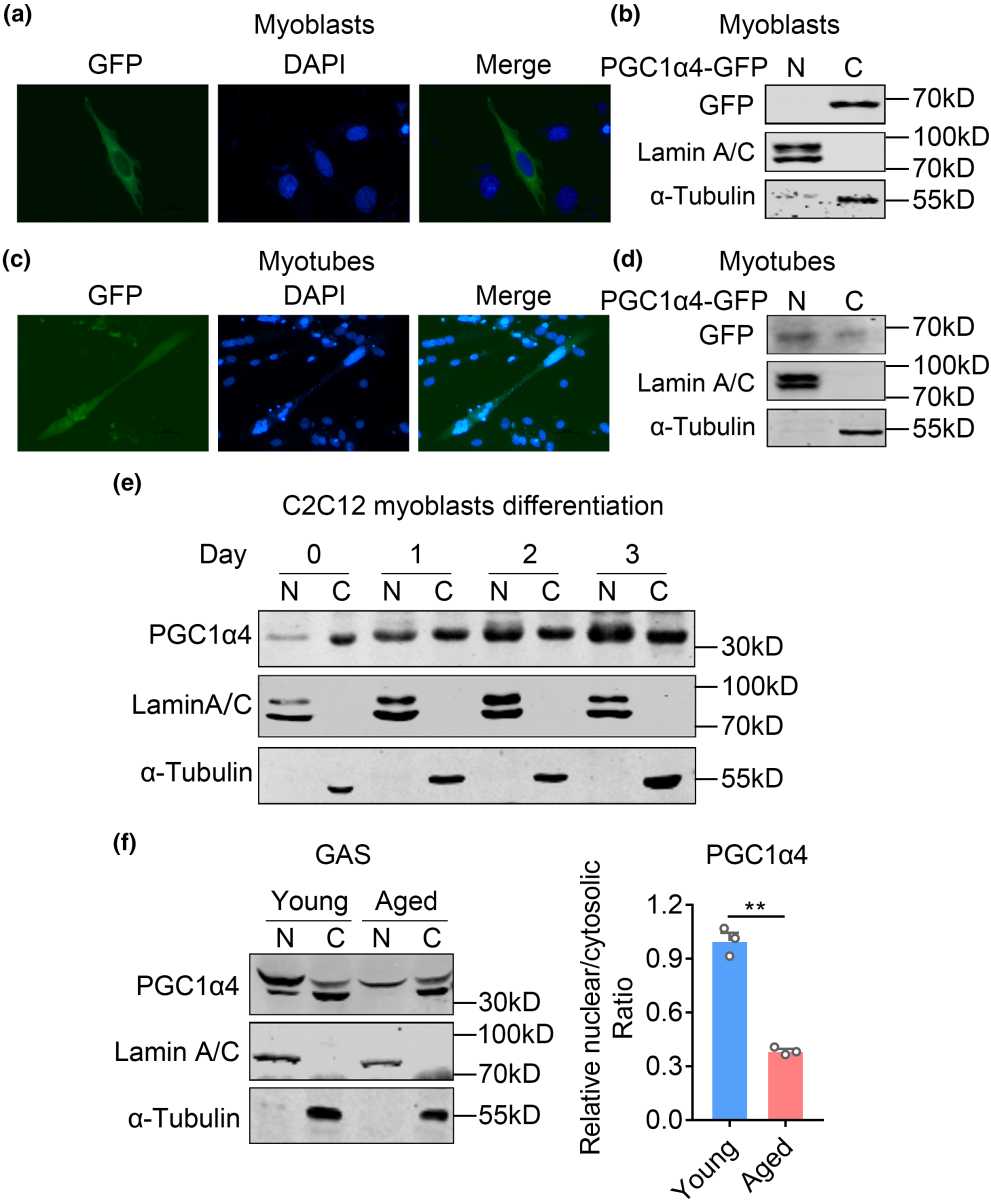
PGC‐1α4 localization in nucleus is reduced in muscle during aging. (a and c) Fluorescence analysis of GFP assessing the localization of PGC1α4‐GFP in C2C12 myoblasts (a) and C2C12 myotubes (c) transfected with GFP fused PGC1α4. Nuclear was stained by DAPI shown as blue. Scale bar = 100 μm. (b, d) Immunoblotting analysis of cytoplasmic (c) and nuclear (N) fractions of C2C12 myoblasts and C2C12 myotubes with GFP antibody. (e) Immunoblotting analysis of endogenous PGC1α4 cytoplasmic (c) and nuclear (N) levels during the differentiation processes from C2C12 myoblasts to myotubes. (f) Immunoblotting analysis (left panel) and relative protein levels (right panel) of PGC1α4 cytoplasmic (c) and nuclear (N) levels in gastrocnemius from young or aged mice. Lamin A/C and α‐Tubulin (bottom) were used as nuclear and cytoplasmic controls, respectively. Data are presented as mean ± SEM and *p < 0.05, **p < 0.01 compared to control group. Scale bar represents 100um.

Interestingly, we performed fluorescence staining and immunoblotting in both C2C12 myoblasts and differentiated myotubes and found that PGC1α4 were dominantly located at cytoplasm in myoblasts, while featured an even distribution in both cytoplasm and nucleus in myotubes (Figures 2a‐d). Of note, using α‐Tubulin and Lamin A/C as cytoplasmic and nucleus control, respectively, immunoblot analysis showed that endogenous PGC1α4 translocated from cytosol to nucleus during muscle cell differentiation, with a gradual increase in nuclear‐localized PGC1α4 proportion along the differentiation time course (Figures 2e), suggesting PGC1α4 plays regulatory roles in myotubes. Moreover, we examined PGC1α4 localization in GAS muscle from young and aged mice and found that in addition to reduced PGC1α4 protein levels as shown in Figure 2b, the nucleus/cytosol ratio of PGC1α4 were also decreased in muscles of aged mice (Figures 2f), suggesting a close correlation of suppressed PGC1α4 expression and nuclear localization with muscle atrophy during aging.

### 2.3 Ectopic expression of NLS‐PGC1α4 increased myotube sizes in vitro

**FIGURE 3:**
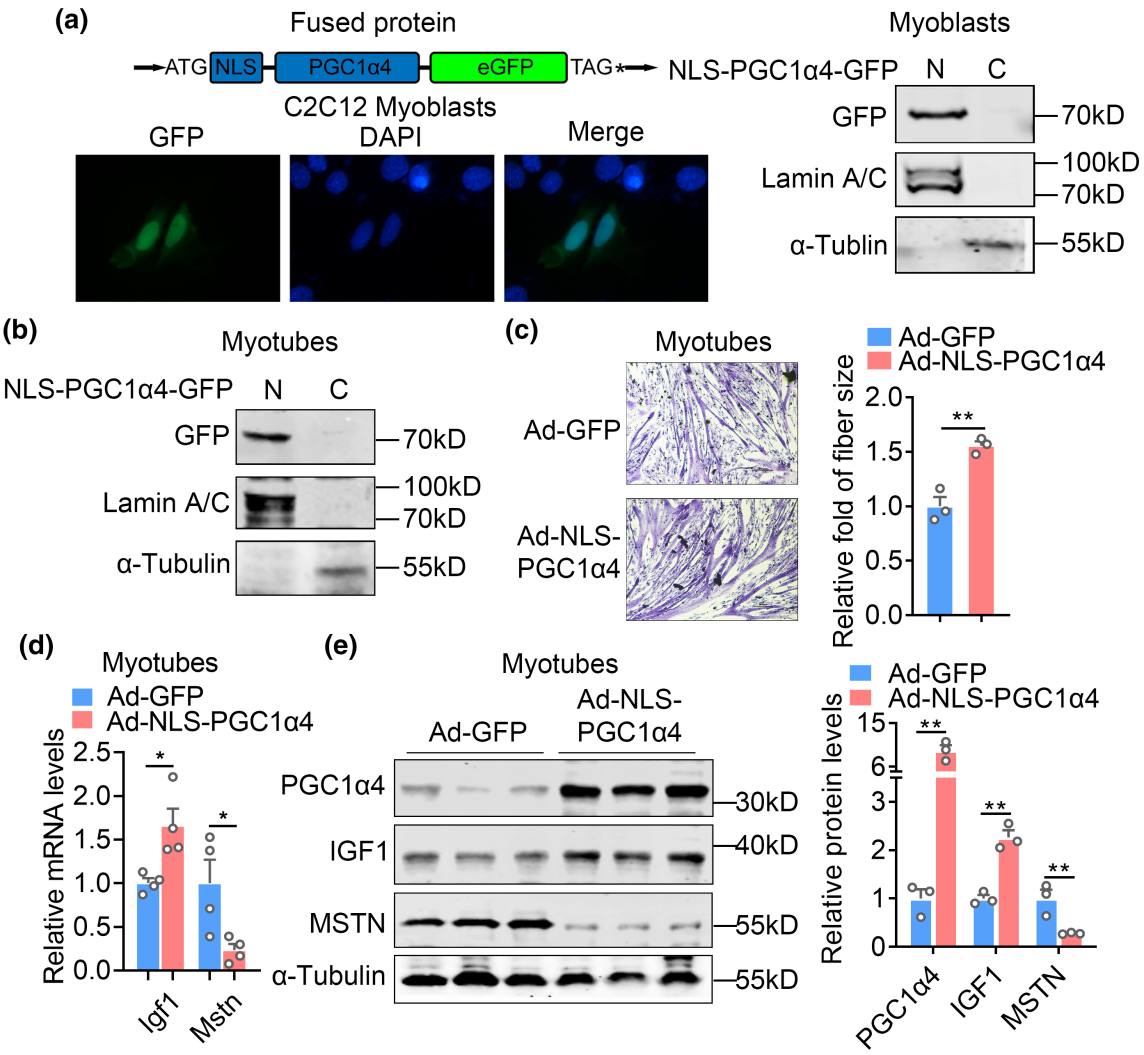
Ectopic expression of NLS‐PGC1α4 increased myotube sizes in vitro. (a, b) Construction strategy of NLS‐PGC1α4‐GFP plasmid, GFP fluorescence and immunoblotting analysis using GFP antibody assessing the localization of NLS‐PGC1α4‐GFP in C2C12 myoblasts or myotubes transfected with GFP fused NLS‐PGC1α4. Scale bar = 100 μm. (c) H&E staining showing fiber sizes of C2C12 myotubes treated with adenoviral delivery system of Ad‐GFP or Ad‐NLS‐PGC1α4‐GFP (short for Ad‐NLS‐PGC1α4) for 48 h and relative fold of myotube diameter were quantified shown as histogram. *n* = 3 per group. Scale bar = 100 μm. (d, e) Real time PCR and protein quantification analysis showed Mstn and Igf1 expression of C2C12 myotubes treated with Ad‐GFP and Ad‐NLS‐PGC1α4 for 48 h. *n* = 4 per group. Data are presented as mean ± SEM and *p < 0.05, **p < 0.01 compared to control group. Scale bar represents 100um.

Given the decline of transcription levels and reduced nuclear localization of PGC1α4 during aging, we designed a NLS‐PGC1α4‐GFP by fusing a 3′ artificial NLS to the N‐terminal of PGC1α4 and GFP to the C‐terminal of PGC1α4 to evaluate whether its ectopic expression could enhance muscle functionality and alleviate sarcopenia (Figure 3a). Of note, fluorescent and immunoblotting analysis revealed that forced expression of NLS‐PGC1α4 increased PGC1α4 protein levels, while featuring a specific nucleus localization in C2C12 myoblasts, myotubes (Figure 3a,b) and HEK293T, NIH3T3 (Figure S3A‐S3C), comparing to the dominant cytosolic location of WT‐PGC1α4 as shown in Figure S1.

Furthermore, we overexpressed control and NLS‐PGC1α4 with adenoviral system in C2C12 myoblasts and subjected them to standard differentiation procedure. Of note, NLS‐PGC1α4 overexpression promoted myotube differentiation and resulted in larger myotube sizes, as well as increased *Igf1* and decreased *Mstn* mRNA and protein levels in differentiated myotubes, compared to controls (Figures 3c–e), suggesting enhanced efficiency of NLS‐PGC1α4 in the promotion of muscle hypertrophy.

We then examined the roles of NLS‐PGC1α4 on myoblast viability, proliferation, and differentiation. Firstly, CCK8 analysis showed that adenovirus mediated NLS‐PGCα4 overexpression in myoblasts increased cell viability (Figure S4A). Secondly, treatment with Adv‐NLS‐PGCα4 induced myoblast proliferation, as shown by enhanced proliferative gene markers such as *Ki67* and cell cycle‐related genes *Cyclin E*, *Cyclin A2*, *Cyclin B2*, *Cdk2*, as well as increased EdU positive cells in EdU fluorescent analysis (Figures S4B, S4C). Thirdly, Adv‐NLS‐PGCα4 increased fusion‐related markers *Myomaker, Myomerger and Caveolin‐3* and differentiation‐related markers *MyoG* and *MyoD*, as well as protein levels of differentiation markers MYOG and MYHC (Figure S4D, S4E), while myotubes treated with Adv‐NLS‐PGC1α4 showed higher fusion and differentiation index as shown by MYHC immunofluorescence staining (Figure S4F). Taken together, these data demonstrated that overexpression of NLS‐PGC‐1α4 induced myoblasts viability, proliferation, and differentiation.

### 2.4 Muscle specific AAV mediated NLS‐PGC1α4 overexpression alleviates aging‐associated sarcopenia in vivo

**FIGURE 4:**
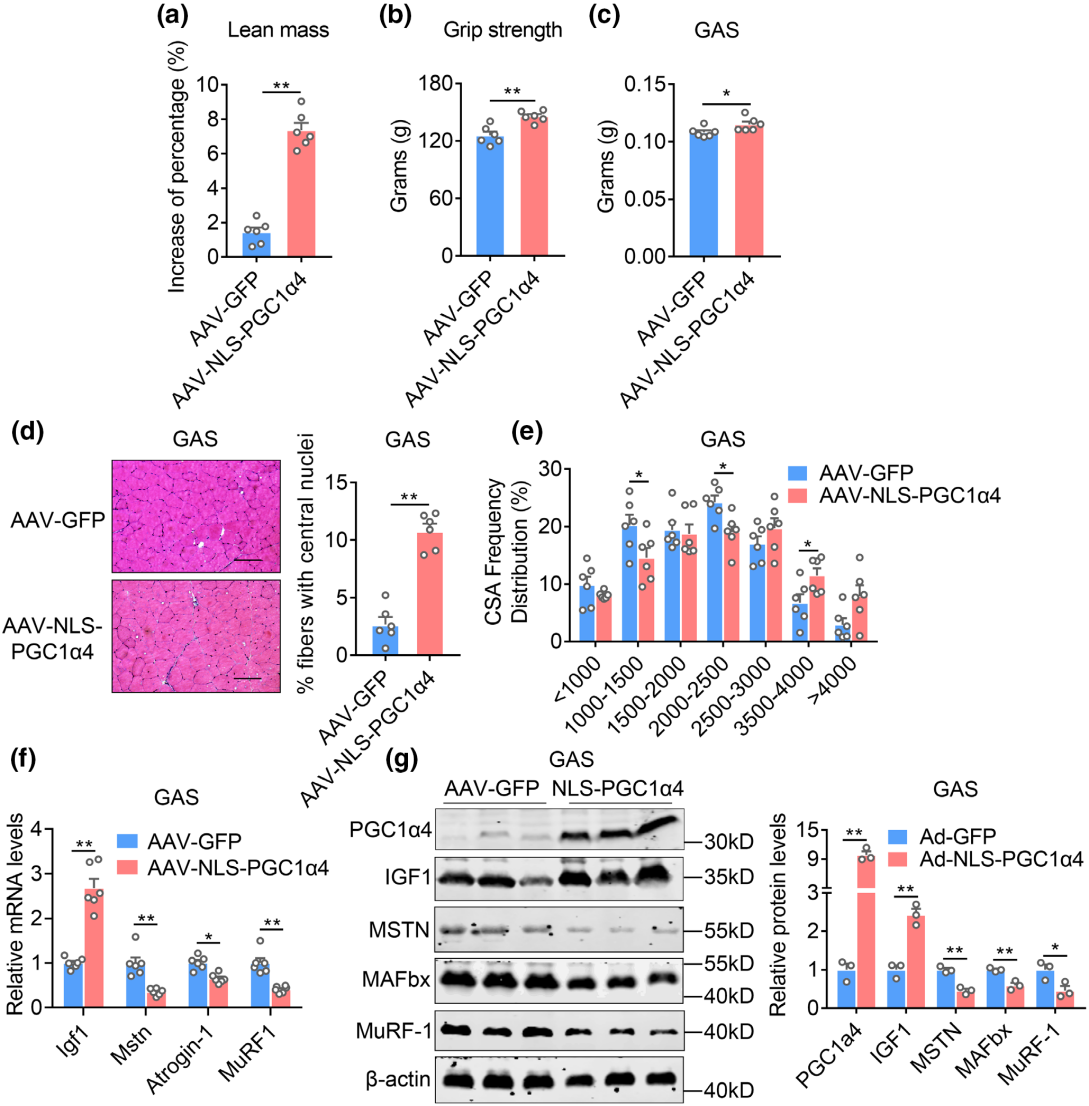
AAV‐mediated NLS‐PGC1α4 ameliorates aging‐induced dysfunction in muscles. (a–g) Phenotypical and molecular analysis of muscles from aged mice (18 months‐old) administrated locally in GAS with AAV delivery system of AAV‐GFP or AAV‐NLS‐PGC1α4‐GFP (short for AAV‐NLS‐PGC1α4) for 8 weeks. *n* = 6 per group. (a) Increase of percentage of lean mass; (b) grip strength; (c) weights of GAS muscles; (d) representative H&E staining and percentage fibers with central nuclei; (e) quantifications of muscle fiber sizes distribution; (f) mRNA expression levels of *Igf1*, *Mstn*, *Atrogin‐1* and *MuRF‐1*; (g) protein levels and quantification of IGF1, MSTN, MAFbx and MuRF‐1 in GAS muscle. Data are presented as mean ± SEM and *p < 0.05, **p < 0.01 compared to control group. Scale bar represents 100um.

To further determine the effects of NLS‐PGC1α4 overexpression in muscle for alleviating aging‐induced sarcopenia in vivo, we adapted an adeno‐associated virus (AAV) system to specifically express a tissue‐specific double muscle creatine kinase (dMCK) promoter (Wang et al., 2008) ‐driven NLS‐PGC1α4 following with GFP in plasmid in mice GAS muscle (Figure S5A). The efficiency and specificity of PGC1α4 overexpression in vivo were then examined. Of note, we observed specific PGC1α4 overexpression in GAS but not in soleus, liver, adipose and heart tissues (Figures S5B‐S5D). We thus injected AAV‐NLS‐PGC1α4 or control (AAV‐GFP) into GAS muscle of aged mice at 18‐month‐old age and performed analysis after 2‐month intervention. Of note, NLS‐PGC1α4 group of aged mice showed a significant increase in lean mass and enhancement in grip strength during a two‐month intervention period (Figure 4a,b). Consistently, GAS muscle weights of NLS‐PGC1α4 group were heavier (Figure 4c) and characterized of larger fiber sizes and fibers with more centrally located myonuclei while similar inflammatory levels (Figure 4d, Figure S4E,S4F). In addition, NLS‐PGC1α4 overexpression exhibited stronger hypertrophic effects on fast type fibers than slow type fibers as shown by MHC I slow fiber immunofluorescence staining (Figure S5G), which were consistent with previous reports that Myo‐PGC1α4 muscle specific PGC1α4 transgenic mice showed a higher representation of type lla and Ilx fibers in muscles (Ruas et al., 2012). Interestingly, NLS‐PGC1α4 drove the appearance of large fibers (>4000 μm^2^ intervals) that were rarely seen in control group (Figure 4e). In addition, AAV mediated NLS‐PGC1α4 overexpression in aged mice resulted in increased *Igf1* and reduced myostatin gene levels, accompanied with reduced muscle atrophy markers *Atrogin‐1* and *MuRF‐1* in their muscles (Figure 4f,g). In summary, these data indicated that AAV mediated muscle specific NLS‐PGC1α4 overexpression promoted muscle hypertrophy and alleviated aging‐induced sarcopenia in mice.

In addition, in order to make parallel comparison between PGC‐1α4 and NLS‐PGC‐1α4, we overexpressed PGC‐1α4 or NLS‐PGC‐1α4 in GAS muscle of aged mice and examined muscle hypertrophic gene programs and muscle fiber sizes compared to GFP control group. We found that PGC‐1α4 itself could induce muscle hypertrophic gene expressions and fiber sizes in aged mice, while NLS‐PGC‐1α4 exhibited stronger effects on these parameters (Figures S6A‐S6D), suggesting the superior efficacy of NLS‐PGC‐1α4 in muscle hypertrophy compared to PGC‐1α4 in aged mice.

### 2.5 NLS‐PGC1α4 overexpression in muscle ameliorates aging‐induced metabolic dysfunction

Skeletal muscle homeostasis is important for systematic metabolic improvements (Pedersen & Febbraio, 2012; Sartori et al., 2021) by increasing insulin sensitivity, reducing lipid dysregulation, enhancing basal metabolic rate and improving hepatic steatosis (Al Saif & Alsenany, 2015; Gundersen, 2011; Rodriguez‐Fdez et al., 2020). In this regard, we evaluated the metabolic consequences of AAV mediated muscle specific NLS‐PGC1α4 overexpression in aged mice.

**FIGURE 5:**
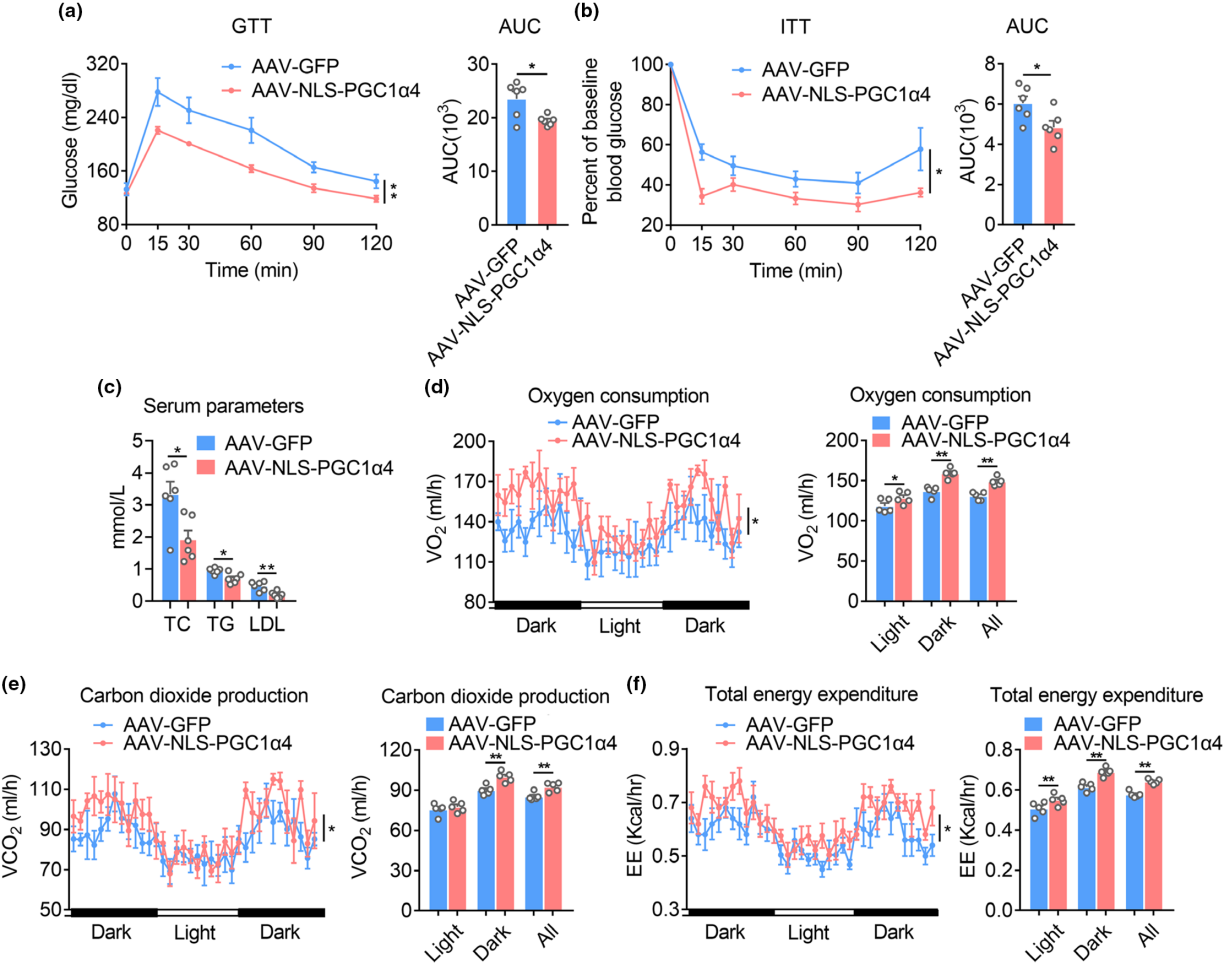
AAV‐mediated NLS‐PGC1α4 ameliorates aging‐induced metabolic dysfunction. (a–f) Metabolic performances of aged mice (18 months‐old) locally administrated with AAV‐GFP or AAV‐NLS‐PGC1α4‐GFP (short for AAV‐NLS‐PGC1α4) in GAS for 8 weeks. *n* = 6 per group. (a) Glucose tolerance test (GTT) and area under the curve (AUC); (b) insulin tolerance test (ITT) and AUC; (c) Serum parameters analysis including total cholesterol (TC), total triglyceride (TG) and low‐density lipoprotein cholesterol (LDL‐C); (d) Oxygen consumption; (e) Carbon dioxide production; (f) Total energy expenditure. (*p* < 0.05, ANCOVA). Statistical significance was assessed by two‐way ANOVA followed with Bonferroni's multiple comparison test (a and b) or unpaired Student's test (c) or ANCOVA with body weight as covariant (d, e, f). Data are presented as mean ± SEM and *p < 0.05, **p < 0.01 compared to control group.

Of note, we found AAV‐NLS‐PGC1α4 group showed enhanced insulin sensitivity as indicated by better performances in glucose and insulin tolerance test compared with control group (Figure 5a,b). Besides, NLS‐PGC1α4 overexpression in GAS of aged mice reduced serum triglyceride, cholesterol and LDL levels, without overt toxicity as demonstrated by unaltered liver and kidney functions (Figure 5c, Table S1). Furthermore, the energy expenditure analysis using CLAMS showed a highly significant increase at oxygen consumption, carbon dioxide production and total energy expenditure in AAV‐NLS‐PGC1α4‐injected mice compared with controls group, while maintaining food intake and locomotor activity (Figure 5d,f and Figures S7A,S7B).

**FIGURE 6:**
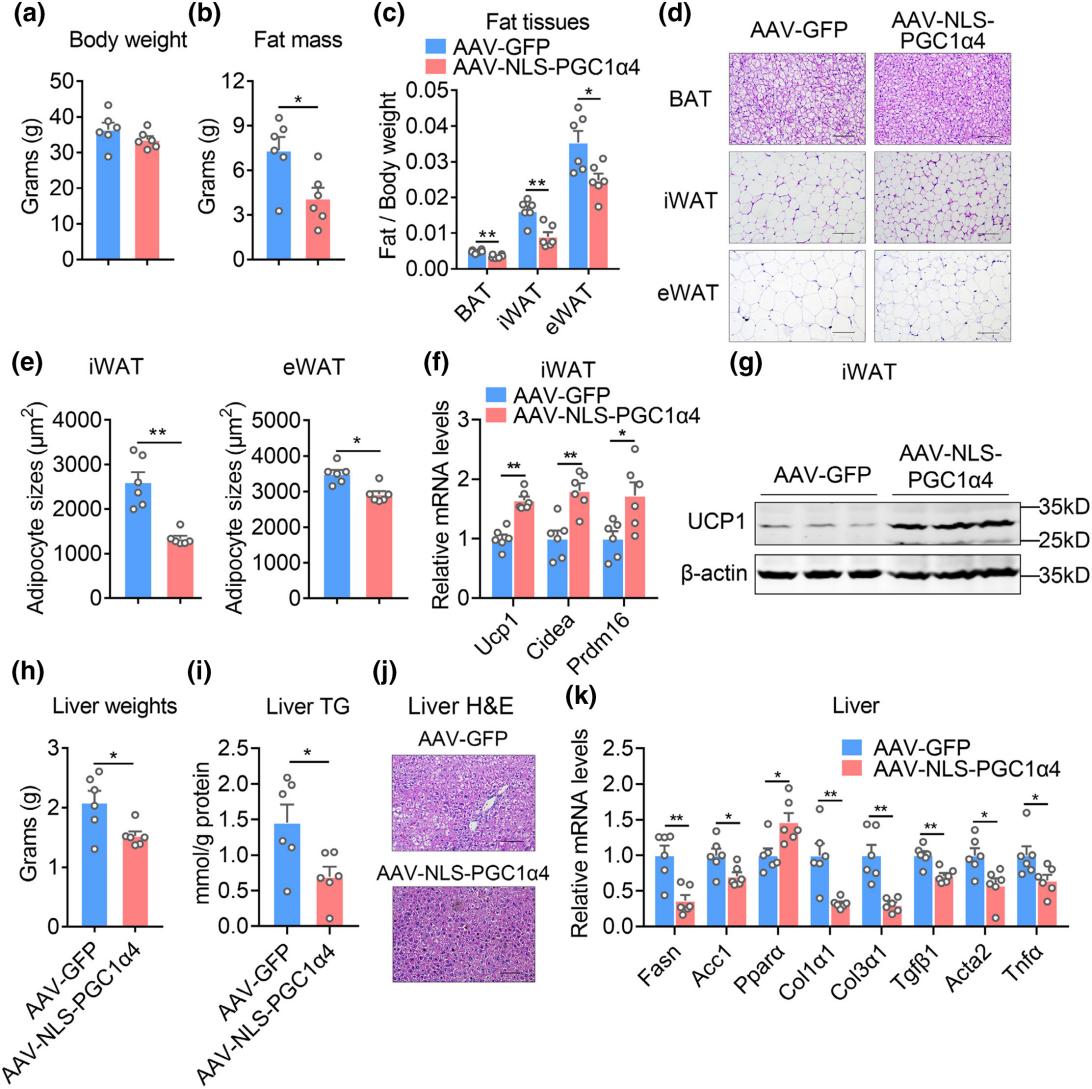
AAV‐mediated NLS‐PGC1α4 improves beige fat functionality and hepatic steatosis. (a–g) Analysis of adipose tissues from aged mice (18 months‐old) locally administrated with AAV‐GFP or AAV‐NLS‐PGC1α4‐GFP (short for AAV‐NLS‐PGC1α4) in GAS for 8 weeks. *n* = 6 per group. (a) Body weight; (b) Fat mass; (c) Fat/Body weight of brown (BAT), inguinal (iWAT), epididymal (eWAT) fat pads; (d) representative H&E staining; (e) quantification of average adipocyte sizes of iWAT and eWAT; (f) mRNA levels of thermogenic genes (*Ucp1, Cidea, and Prdm16*); (g) protein levels of UCP1 in iWAT. (h–k) Analysis of liver tissues from aged mice (18 months‐old) locally administrated with AAV‐GFP or AAV‐NLS‐PGC1α4 in GAS for 8 weeks. *n* = 6 per group. (h) liver weights; (i) hepatic TG contents; (j) representative H&E staining; (k) mRNA levels of hepatic gene programs related to β‐oxidation and inflammation in liver. Data are presented as mean ± SEM and *p < 0.05, **p < 0.01 compared to control group. Scale bar represents 100um.

Detailed analysis revealed that, although no body weight changes were observed in AAV‐NLS‐PGC1α4 group, possibly due to increased lean mass and reduced fat mass (Figure 6a,b), QU and TA muscles were heavier with enlarged fiber sizes after NLS‐PGC1α4 overexpression in GAS muscle (Figures S7C‐S7F). Besides, AAV‐NLS‐PGC1α4 group of mice displayed fewer fat weights in BAT, iWAT, and eWAT tissues (Figure 6c), accompanied with smaller fat depots and average adipocyte sizes (Figure 6d,e), as well as increased thermogenic gene programs (*Ucp1, Cidea, Prdm16*) in iWAT, compared to AAV‐GFP control group (Figure 6f,g). Furthermore, AAV‐NLS‐PGC1α4 group featured less liver weights, hepatic lipid infiltration, triglyceride levels, as well as decreased lipid synthesis (*Fasn, Acc1*) and increased β‐oxidative gene (*Pparα*), reduced fibrotic genes (*Col1α1, Col3α1*) and proinflammatory cytokines (*Tgfβ1, Acta2, Tnfα*) compared with control group (Figures 6h–k). Overall, these data suggested that NLS‐PGC1α4 overexpression specific in GAS muscle of aged mice not only alleviated sarcopenia in other muscle tissues, but also ameliorated aging‐associated metabolic dysfunctions, thus represented a potent and safe strategy for healthy aging.

### 2.6 NLS‐PGC1α4 overexpression in GAS muscle improved muscle and adipose tissue functionality and systemic energy metabolism in aged mice

**FIGURE 7:**
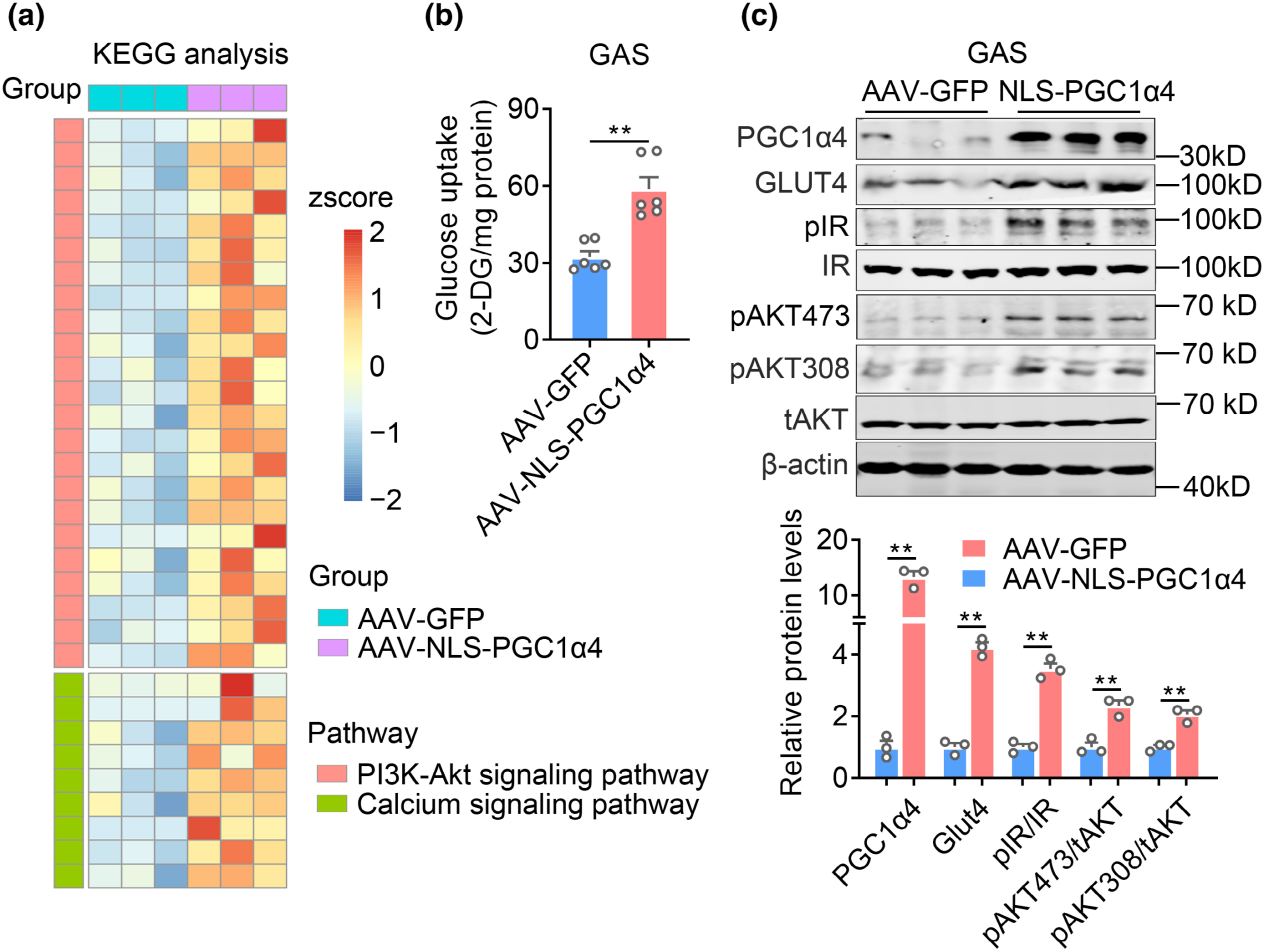
NLS‐PGC1α4 delivery in GAS muscle enhances glucose uptake and improves insulin signaling in skeletal muscle. (a–c) Analysis of aged mice locally administrated with AAV‐GFP or AAV‐NLS‐PGC1α4 in GAS. *n* = 6 per group. (a) Heatmap showing the differential genes in PI3K‐Akt signaling pathway and calcium signaling pathway by KEGG analysis; (b) The glucose uptake in gastrocnemius muscle; (c) The protein levels of PGC1α4, GUT4, pIR, IR, pAKT473, pAKT308, tAKT, β‐actin, and quantification. Data are presented as mean ± SEM and *p < 0.05, **p < 0.01 compared to control group.

To further elucidate the molecular mechanisms of NLS‐PGC1α4 against sarcopenia and identify its possible target genes in aged muscles, we performed RNA‐seq analysis on GAS muscle treated with control or NLS‐PGC1α4. KEGG analysis highlighted PI3K‐Akt and calcium signaling pathways (Figure 7a), which are closely related to skeletal muscle insulin sensitivity and hypertrophy (Glass, 2010; Sakuma & Yamaguchi, 2010), were significantly enhanced in AAV‐NLS‐PGC1α4 group. Consistently, in addition to muscle hypertrophy, AAV‐NLS‐PGC1α4 increased glucose uptake (Figure 7b), as well as protein levels of glucose transporter GLUT4 and insulin signaling cascades including phosphorylated IR and AKT (Thr308 and Ser473) in skeletal muscle compared with AAV‐GFP (Figure 7c).

**FIGURE 8:**
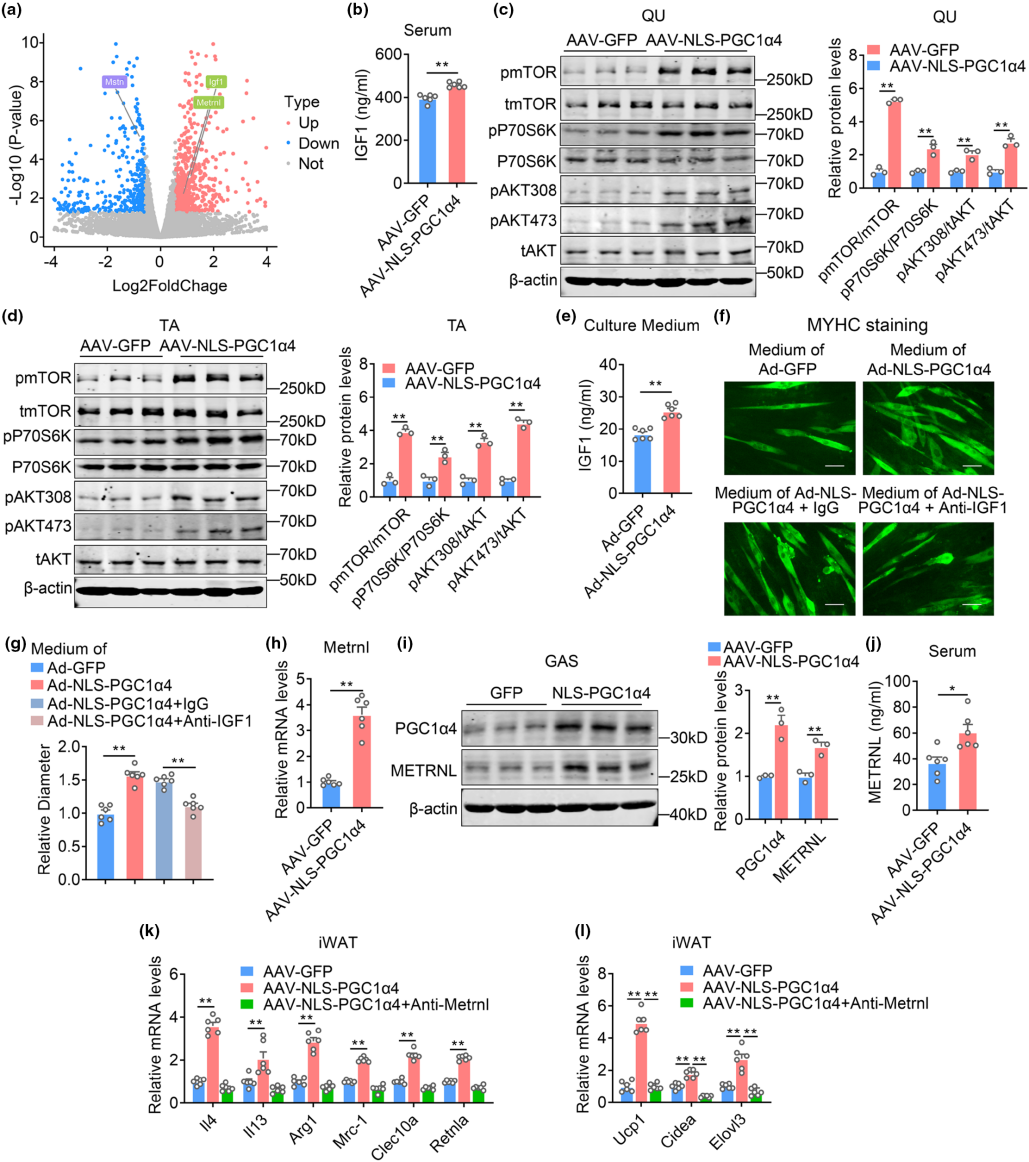
IGF1 and METRNL mediates beneficial effects of NLS‐PGC1α4 on muscle and adipose tissue functionality in aged mice. (a–d) Analysis of aged mice locally administrated with AAV‐GFP or AAV‐NLS‐PGC1α4 in GAS. *n* = 6 per group. (a) Volcano plot showing the differential genes in gastrocnemius muscle between AAV‐NLS‐PGC1α4 and AAV‐GFP group mice; (b) The serum METRNL levels; (c, d) The protein levels of pmTOR, tmTOR, pP70S6K, P70S6K, pAKT308, pAKT473, tAKT, β‐actin and quantifications in QU and TA muscles. (e) IGF1 protein contents in culture medium from myotubes treated with Ad‐GFP or Ad‐NLS‐PGC1α4; (f, g) Representative MYHC immunofluorescence staining and relative diameter of myotubes treated with culture medium from Ad‐GFP or Ad‐NLS‐PGC1α4 with or without Anti‐IGF1 neutralizing antibody treated myotubes. (h, i) The mRNA levels of *Metrnl* and the protein levels and quantification of PGC1α4, METRNL and β‐actin in GAS muscles; (j) METRNL protein contents in serum of aged mice locally administrated with AAV‐GFP or AAV‐NLS‐PGC1α4‐GFP in GAS; (k, l) The mRNA levels of *Il4*, *Il13*, M2 macrophage genes *Arg1*, *Mrc1*, *Clec10a*, *Retnla* (k) and thermogenic genes *Ucp1, Cidea, Elovl3* (l) in iWAT from aged mice locally administrated with AAV‐GFP or AAV‐NLS‐PGC1α4‐GFP in GAS and followed by intraperitoneal injection (i.p.) of Anti‐Metrnl neutralizing antibody every other day for 2 weeks.

Since overexpression of NLS‐PGC1α4 in GAS muscle exhibited strong systematic effects, we focused on secreted factors that were largely altered in the RNA‐seq dataset. Of note, we noticed that the well‐studied secreted myokines Igf1 and Metrnl were increased, while Mstn was decreased by NLS‐PGC1α4 administration, which were confirmed by qPCR analysis (Figure 8a). IGF1 has been well documented to increase muscle hypertrophy via IGF1‐AKT–mTOR signaling pathway (Rommel et al., 2001; Yoshida & Delafontaine, 2020). Importantly, in AAV‐NLS‐PGC1α4 GAS overexpression mice, we found increased mRNA and protein levels of Igf1 in GAS muscle, while serum IGF1 levels were increased (Figure 8b) and IGF1‐AKT–mTOR signaling pathway in QU and TA muscles including phosphorylated AKT (Thr308 and Ser473), phosphorylated mTOR (Ser2448), phosphorylated S6K (Thr389) were dramatically enhanced (Figure 8c and d), suggesting NLS‐PGC1α4 administration in GAS may exert hypertrophic effects in other muscles via IGF1 secretion. Indeed, Ad‐NLS‐PGC1α4 treatment in myotubes increased IGF1 concentrations in culture medium (Figure 8e). Immunofluorescence analysis showed that myotubes treated with culture medium from Ad‐NLS‐PGC1α4 cells exhibited enlarged fiber sizes, which were blocked by IGF1 neutralizing antibody treatment (Figure 8f,g). These data suggested that NLS‐PGC1α4 administration in GAS muscle may enhance muscle hypertrophy and exert systemic protection against aging‐associated sarcopenia at least partially via IGF1.

Besides, among myokines featured enhanced levels in RNA‐seq, METRNL attracted our attention since it has been shown to regulate skeletal muscle regeneration and browning of white fat (Rao et al., 2014). Notably, in AAV‐NLS‐PGC1α4 GAS overexpression mice, we found enhanced Metrnl mRNA and protein levels in GAS muscle, as well as increased METRNL levels in serum (Figure 8h–j), suggesting METRNL may serve as a mediator for systematic metabolic homeostasis. METRNL has been reported to activate inguinal fat tissue thermogenesis by inducing IL4/IL13 and activating M2 macrophages (Li et al., 2023). Indeed, we found that reported METRNL target genes *Il4*, *Il13*, *Arg1*, *Mrc1*, *Clec10a*, and *Retnla* were induced in iWAT after NLS‐PGC1α4 overexpression in GAS (Figure 8k). Metrnl neutralizing antibody treatment abrogated the induction of these genes, in the meantime suppressed thermogenic gene expression in iWAT (Figure 8k,l). These data suggested that NLS‐PGC1α4 overexpression in GAS muscle led to browning of white fat at least partially via METRNL.

Overall, considering that PGC1α4 is a transcriptional cofactor, its overexpression and nuclear retention in GAS muscle via NLS‐PGC1α4 may cooperate with multiple key transcription factors to govern expressions of myokines, such as Igf1 and Metrnl, which in turn crosstalk with other muscles and fat tissues for systemic improvement in energy homeostasis.

## 3 DISCUSSION

In the present study, we found the transcriptional expression levels and nucleus protein levels of PGC1α4 were reduced in muscles of sarcopenic mice. Thus, we designed and successfully applied AAV mediated muscle‐specific muscle creatine kinase (MCK) promoter‐driven NLS‐PGC1α4 in GAS muscle and alleviated aging‐associated sarcopenia and metabolic disorders, including adiposity, insulin resistance, and fatty liver. Mechanistically, we found that NLS‐PGC‐1α4 improved insulin signaling and enhanced glucose uptake in skeletal muscle. Besides, RNA‐seq analysis identified IGF1 and METRNL as potential targets of NLS‐PGC‐1α4 that possibly mediate the improvement of muscle and adipose tissue functionality in aged mice. In addition, we showed clinical relevance of PGC‐1α4 and sarcopenia. Overall, these data suggested NLS‐PGC1α4 as a potent therapeutic strategy in combating aging‐associated sarcopenia and metabolic dysfunctions.

Aging‐associated sarcopenia is characterized of muscle mass loss and functionality decline. It can often lead to impaired mobility and adverse metabolic consequences such as insulin resistance, reduction of basal metabolic rate and increases in fat mass (Petrocelli & Drummond, 2020; Robinson et al., 2017; Tezze et al., 2017). Although exercise is beneficial to improve sarcopenia (Colleluori et al., 2019). it is not applicable to all elders. Of note, PGC1α4 could be served as a rational target to improve sarcopenia since it has been shown to prevent or treat muscle wasting during muscle injury or cancer related cachexia by engaging multiple beneficial mechanisms for muscle hypertrophy, that is, IGF1 induction, myostatin inhibition, and increased protein synthesis via mTOR (Ruas et al., 2012; White et al., 2014). In addition, PGC1α4 is the short isoform of PGC1α with 266 amino acids, thus could be easily packaged in AAV to achieve a relatively safe and high transfer capacity.

We found that during aging, PGC1α4 transcription is reduced in GAS muscle, which is possible due to decreased CREB phosphorylation in aging. Consistently, it has been reported that aged mice demonstrated lower Pgc1α4 mRNA in aging‐induced atrophic TA muscle via MAPK signaling (Brown et al., 2017). CREB is a downstream effector of MAPK and contributes to muscle hypertrophic growth, mitochondrial biogenesis, metabolic efficiency, and muscle performance (Berdeaux & Hutchins, 2019). Thus, the decreased CREB phosphorylation‐Pgc1α4 transcriptional axis in muscle during aging might be responsible for sarcopenia.

In addition, via bioinformatic analysis and cellular verification, we found that PGC1α4 lacks nuclear localization sequence and expressed dominantly in cytosol in HEK293T, NIH3T3, and C2C12 myofibroblasts, while translocated into nuclear under myotube differentiation. This nuclear translocation phenomenon is also reported in hepatocytes following addition of TNFα (Leveille et al., 2020). The mechanism of PGC1α4 translocation is still not clear. It is possible that PGC1α4, as a cofactor, could be recruited by other transcription factors and translocate into nucleus in response to specific stimuli. Further work with PGC1α4 Co‐IP mass spectrometry would provide more information to reveal this translocation mechanism. Nevertheless, considering that PGC1α4 majorly exert its function in nuclear, we design an artificial NLS to fuse with PGC1α4 and promote its localization in nuclear, which achieved enhanced muscle hypertrophy both in vitro and in vivo.

It is interesting that immunoblotting assay showed one major band with exogenous NLS‐PGC1α4 overexpression, while two bands of endogenous PGC1α4 in in vivo samples (Figures 1f and 2f), compared to one band in in vitro immunoblotting data (Figure 2e), suggesting that endogenous PGC1α4 may undergo protein modifications in vivo. Based on structure analysis and previous reports on different PGC1α isoforms, PGC1α4 may undergo various post‐transcriptional modification, that is, Sumoylation or phosphorylation (Puigserver et al., 2001), which may cause the two bands observed in immunoblots in vivo. As different post‐transcriptional modification changes protein localization (Miller et al., 2019), it is possible that in GAS of aged mice, nuclear‐localized PGC1α4 featured specific modification, which may result in its unique band form we observed in this study. The characteristics of PGC1α4 protein modification and function in aging warrant further investigation.

In recent years, AAV mediated gene therapies have been used to treat various diseases in pre‐clinical and clinical studies due to its safeness and effectiveness. Among multiple types of AAVs, AAV9 is one of the most widely used vectors for treating muscle diseases for its high delivery efficiency in muscle (Bulaklak et al., 2018; Lim et al., 2020; Nance et al., 2019). It has been shown that AAV9 mediated gene delivery, such as miR‐23a/27a, significantly protected against loss of muscle force and reversed muscle dystrophic features in rodents (Zhang et al., 2018). In addition to rodents, AAV9‐mediated gene therapies have been shown to improve muscle histology in young adult Duchenne muscular dystrophy dogs (Yue et al., 2015) Besides, AAV9 is chosen in mouse models for treating cardiac muscle diseases such as Brugada syndrome, arrhythmias, and mild cardiomyopathy (Yu et al., 2022). In order to specifically target muscle cells in vivo, we designed a MCK promoter to ensure specific expression in muscle. Moreover, we further utilized local muscle injection to specifically deliver AAV‐NLS‐PGC1α4 in muscle to avoid systematic impacts. Indeed, we did not observe increased PGC1α4 in adipose tissues, livers or hearts, and the liver and kidney functions were unaltered, suggesting the effectiveness and safeness of our delivery strategy against sarcopenia and aging‐associated metabolic diseases. It is recently reported that MyoAAV with a class of RGD motif‐containing capsids transduces muscle with high efficiency and selectivity (Tabebordbar et al., 2021). Future work on other AAV delivery system is warranted.

Exercise strengthens skeletal muscle and increases whole body energy expenditure. Of note, we demonstrated systematic improvements on muscle mass, insulin sensitivity, lipid parameters, fat mass, and hepatic steatosis after AAV‐NLS‐PGC1α4 delivery in muscle. Although in our study, the NLS‐PGC1α4 is expressed in GAS, the phenotypes we observed were consistent with the phenotypes of Myo‐PGC1α4 muscle specific PGC1α4 transgenic mice (Rao et al., 2014). This local overexpression of PGC1α4 induced significant improvements on systemic metabolism may be mediated by various mechanism. For example, PGC1α4 increases anaerobic glycolysis in a PPARβ‐dependent manner and promotes muscle glucose uptake and fat oxidation (Koh et al., 2022). Moreover, the nuclear‐localized form of PGC1α4 may have more potent transactivation ability due to its high localization in nuclear. We studied the functions of NLS‐PGC1α4 in aged mice characterized of sarcopenia and metabolic dysfunction, which might also potentiate the PGC1α4‐mediated whole‐body metabolic improvement. Furthermore, we demonstrated that NLS‐PGC1α4 overexpression in GAS may exert systematic effects in muscle function and energy homeostasis by its governing on the expression of circulating factor IGF1 and meteorin‐like (Metrnl), which have been shown to promote muscle hypertrophy and increases thermogenesis and anti‐inflammatory gene programs in fat via alternative activation of adipose tissue macrophages, respectively (Rao et al., 2014; Rommel et al., 2001). It would be interesting to perform additional loss‐of‐function studies and systemically elucidate the mechanism for PGC1α4‐mediated metabolic improvements and reduction in adiposity.

In summary, based on our finding that PGC1α4, a newly defined potent regulator for muscle hypertrophy, were reduced in both transcription level and nuclear localization in skeletal muscle of aged mice, we designed and delivered a AAV9 mediated and MCK driven nuclear localized PGC1α4 in GAS muscle of aged mice and resulted in significant improvements in muscle functionality and systematic metabolic performances, which represents a potent strategy against sarcopenia and aging‐associated metabolic disorders.

## 4 MATERIALS AND METHODS

### 4.1 Animal assays

C57BL/6J male mice were purchased from Shanghai Laboratory Animal Center (Shanghai, China). All animal experiments and protocols were conducted under the ethical guidelines of the East China Normal University. Mice were maintained in standard 12‐h light/12‐h dark cycle and had free access to food and water. All mouse studies were approved by the Animal Ethics Committee of East China Normal University. Body composition was measured by AccuFat‐1050 NMR system (MAGMED) and body weight was recorded weekly. Oxygen and Carbon dioxide consumption were recorded via CLAMS system (Columbus instruments system). A digital grip‐strength meter (BIOSEB Research Instruments, BIO‐GS3) was used to measure the limb muscular strength of mice as previously reported (Li et al., 2020). Briefly, the mice were acclimatized to the meter for 10 min before the grip strength test began. The legs of mice with virus injected were permitted to grab the metal pull bars and were pulled backwards by the tail. The force at the time of release was recorded as the peak tension. Each mouse was tested five times with a 30 s break and the average data was used for statistical analysis. Investigators were blind to the animal groups.

### 4.2 Cell lines and culture

C2C12 cells (mouse skeletal myoblasts), NIH3T3 (mouse Embryonic fibroblast) and 293 T cells (human embryonic kidney) were obtained from the ATCC and cultured in Dulbecco's modified Eagle's medium (DMEM) with 10% fetal bovine serum and 1% penicillin and streptomycin at 37°C supplemented with 5% CO2. For C2C12 differentiation, cells were planted on culture plates covered with 0.1% gelatin, and when cell confluence reached 70%, the medium was switched into differentiation medium (DMEM containing 2% horse serum) for 4 days. The C2C12 myoblasts were transduced with indicated adenovirus (Ad‐GFP or Ad‐NLS‐PGC1α4‐GFP, short for Ad‐NLS‐PGC1α4, Shanghai Genechem) at multiplicity of infection (MOI) of 100 with 48 h and differentiated into myotubes for in vitro molecular analysis.

For the collection of conditioned medium, the media of cultured myotubes treated with Ad‐GFP or Ad‐NLS‐PGC1α4 for 48 h were collected. For cocultured and neutralizing antibody assays, mouse IGF1 (4 μg/mL) neutralizing antibodies (R&D Systems, AF791) or IgG (Beyotime, A7028) were added into cultured media consists of conditioned medium and differentiation medium mixed with 1:1 ratio (vol./vol.). The media were replaced every 2 days for a total of 6 days and followed by immunofluorescence staining. For differentiation analysis, the myotubes were stained with antibodies against MYHC (Developmental Studies Hybridoma Bank, AB2147781).

### 4.3 CCK‐8 and EdU analysis

The C2C12 myoblast cells were cultured in 96 wells and treated with Ad‐GFP or Ad‐NLS‐PGC‐1α4 for 24 h and analyzed with proliferative capacity with CCK‐8 assay (Beyotime, C0043) or EdU Cell Proliferation Kit with Alexa Fluor 594 (Beyotime, C0078S) following manufacturers' instructions.

### 4.4 Plasmid constructions, transfections, luciferase assays, and fluorescence

PGC‐1α4 promoter was cloned into PGL4.17 vector. The NLS was 5’‐CCTAAGAAAAAGAGGAAGGTG‐3′ and the Pgc1α4 gene was amplified from muscle cDNA by PCR. NLS‐PGC1α4 protein was engineered with three repeats of NLS at N terminus of PGC1α4 (Zhou et al., 2018). The PGC‐1α4, NLS‐PGC‐1α4, CREB, mutated CREB expression plasmids were cloned into pCDH vector. The primers used for plasmids construction were listed in Table S2. HEK293T cells were transfected with indicated plasmids with EZ‐trans transfection reagent (Life Ilab Biotechnology, C4058L1090). Luciferase activity was examined with dual luciferase system (Promega, TM040) after plasmids transfection for 24 h. For examination of PGC1α4 and NLS‐PGC1α4 location, C2C12 myoblasts and myotubes were transfected with GFP fused PGC1α4 or NLS‐PGC1α4 by EZ‐trans transfection reagent (Life Ilab Biotechnology, C4058L1090) and visualized with GFP fluorescence. AAV‐NLS‐PGC‐1α4 were driven by dMCK promoter, while NLS‐PGC1α4 is separated from GFP with a cleavage protein T2A linker.

### 4.5 Real time PCR

Total RNA was extracted from whole GAS muscle tissues (including red GAS and white GAS) or cells using RNAiso Plus (Takara, 9108) following manufacturer's instructions. 1 μg of total RNA was reverse transcribed into cDNA using the PrimeScript™ RT Master Mix (TaKaRa, RR036A). Quantitative real time PCR was performed with the Roche LightCycler 480 system (Roche) using SYBR Green Master mix (Yeasen, 11143ES50). The mRNA levels were calculated by the 2^‐ΔΔCT^ method with the level of GAPDH as the internal control. The primers used for real time PCR were listed in Table S2.

### 4.6 Western blots

The whole GAS muscle (including red GAS and white GAS) were homogenized and cells were lysed in radioimmunoprecipitation (RIPA) buffer containing 50 mM Tris (pH 7.4), 150 mM NaCl, 1% Triton X‐100, 1% sodium deoxycholate, 0.1% SDS, sodium orthovanadate, sodium fluoride, EDTA, leupeptin, supplemented with 1 mM PMSF, 10 mM DTT, and 10 μM protein kinase inhibitor on ice for 5 min. The nuclear and cytoplasmic proteins from cells were separated by a commercialized extraction kit (Beyotime) following the manufacturer's instructions. The protein concentrations were quantified using a BCA Protein quantification kit (Beyotime) and equal amounts of protein were subjected to SDS‐PAGE electrophoresis and transferred onto nitrocellulose (NC) membranes. Membranes were blocked with 5% skimmed milk for 1 h at room temperature and then incubated with primary and secondary antibodies. The densitometry of the western blots was detected by the Odyssey imaging system (LI‐COR Biotechnology). The primary antibodies used were as follows: Anti‐GFP (Proteintech, 50,430‐2‐AP), Anti‐PGC‐1α4 (against N‐terminus of PGC‐1α, Santa Cruz, sc‐518,025), Anti‐P‐CREB (Santa Cruz Biotechnology, sc‐81,486), Anti‐METRNL (Shanghai Kanglang Biotechnology, bs‐18810R), Anti‐MYHC (DSHB, AB2147781), Anti‐MYOG (Beyotime, AF7542), Anti‐GLUT4 (Beyotime, AF6999), Anti‐pIR (CST, 3024), Anti‐IR (ABclonal, A19067), Anti‐pAKT‐Thr308 (CST, 2965S), Anti‐pAKT‐Ser473 (CST, 4060S), Anti‐total‐AKT (Beyotime, AA326), Anti‐pmTOR‐Ser2448 (CST, 2971S), Anti‐total‐mTOR (Beyotime, AM832), pP70S6K‐Thr389 (CST, 9205S), Anti‐total‐P70S6K (CST, 9202S), Anti‐IGF1 (Santa Cruz Biotechnology, sc‐74,116), Anti‐MSTN (Santa Cruz Biotechnology, sc‐393,335), Anti‐MAFbx (Santa Cruz Biotechnology, sc‐166,806), Anti‐MuRF‐1 (Santa Cruz Biotechnology, sc‐398,608), Anti‐β‐actin (Santa Cruz Biotechnology, sc‐8432), Anti‐Lamin A/C (Beyotime, AF7350), and Anti‐α‐Tubulin (Beyotime, AF0001).

Of note, the PGC1α4 antibody (Santa Cruz, sc‐518,025) was purchased as previously reported (Ruas et al., 2012). For verification of antibody specificity, we treated HEK293T cells with Adv‐NLS‐PGC1α4 for 48 h to induce protein expression and stained samples of volume gradient with the antibody. Immunoblotting analysis showed that the anti‐PGC1α4 antibody specifically recognize PGC1α4 band at 34 kDa in a dose dependent manner, which indicated that the antibody specifically recognizes PGC1α4 (Figure S8).

### 4.7 in vivo chromatin immunoprecipitation assays

ChIP experiments were performed using a Simple ChIP Enzymatic Chromatin IP kit (no.9003; Cell Signaling Technologies) according to manufacturer instructions. The fresh GAS from 8‐week‐old C57BL/6J mice were cut with scissors and then cross‐linked with 37% formaldehyde at a final concentration of 1% at room temperature for 10 min. Fragmented chromatin was treated with nuclease and subjected to sonication. Samples were then precleared with protein‐A/G sepharose beads and immunoprecipitated with anti‐p‐CREB‐1 (10E9) antibody (sc‐81,486; Santa Cruz Biotechnology), anti‐normal mouse IgG (sc‐2025; Santa Cruz Biotechnology) as a negative control overnight at 4°C. Chromatin protein/DNA complexes were eluted from the agarose beads by adding 100 μL of elution buffer at room temperature and heated to 65°C for 4 h. After reverse cross‐linking and DNA purification, immunoprecipitated DNA was quantified by real time PCR using power SYBR green (Catalog no. 4367659; Applied Biosystems) with primers for CREB binding sites in the Pgc1α4 promoter. Fold enrichment was calculated based on the threshold cycle (CT) value of the IgG control using the comparative CT method. Primers used in this assay are listed in Table S2.

### 4.8 AAV virus packaging and local injection into muscles

We generated AAV‐dMCK‐NLS‐PGC‐1α4‐GFP plasmid, in which a skeletal muscle‐cell‐specific dMCK promoter is used to drive the expression of PGC‐1α4 in skeletal muscle, following with GFP in plasmid (Wang et al., 2008). AAV‐EGFP was used as control. AAV9 virus were packaged by Hanbio Techonology Company (Shanghai, China). For local intramuscular AAV delivery, 18 months old male mice were randomized into AAV‐NLS‐PGC1α4‐GFP group (short for AAV‐NLS‐PGC1α4, *n* = 6) or AAV‐GFP group (*n* = 6) and anesthetized deeply with isoflurane. The mice were injected intramuscular to bilateral GAS muscles at the dose of 5*10^10^ vector genome (vg) in a final volume of 50 μL per GAS (10^11^ vg/mice). The mice were sacrificed and analyzed after 2‐month intervention. For Metrnl neutralization in vivo, 24 months old male mice were injected with AAV intramuscularly to bilateral GAS muscles for 1 month followed by 5 μg of Anti‐Metrnl or IgG control antibody diluted in saline to a total volume of 200 μL and injected intraperitoneally (i.p.) every other day for 2 weeks. The mice were sacrificed and analyzed for iWAT gene program.

### 4.9 Lentivirus local injection into muscles

For lentivirus local injections in muscle, 24 months old male mice were randomized into Lenti‐NLS‐PGC1α4, Lenti‐PGC1α4 or Lenti‐GFP group and anesthetized with isoflurane. The mice were injected with lentivirus intramuscularly to bilateral GAS muscles at the dose of 10^8^ transducing units (TU) in a final volume of 50 μL per muscle. The mice were sacrificed and analyzed after 1‐month intervention.

### 4.10 Glucose and insulin tolerance tests (GTT and ITT)

For insulin tolerance tests, mice received an intraperitoneal injection of insulin (0.75 U/kg, Sigma, I9287). For glucose tolerance tests, mice were fasted 6 h and injected intraperitoneally with a glucose solution in PBS (1.5 g/kg, Sigma, G8270). Plasma glucose levels were measured from tail vein blood at 0, 15, 30, 60, 90, and 120 min after insulin or glucose injections, using an automatic glucometer (OneTouch Ultra, Johnson's). Area under the curve (AUC) was calculated by GraphPad software.

### 4.11 Histological analysis

Muscle tissues were freshly isolated from mice, flash frozen in OCT and cold isopentane, and cut at 10 μm per section. Frozen sections were placed in 4% PFA for subsequent H&E staining. 5 μm paraffin sections were prepared from fat and liver tissues for subsequent H&E staining. Tissue sections were photographed using Nikon camera. For IF staining, muscle fibers were stained with antibody against MHC I (BA‐D5, Developmental Studies Hybridoma Bank) or MYHC (AB2147781, Developmental Studies Hybridoma Bank). Briefly, myotubes were fixed in ice‐cold 4% PFA for 15 min and 0.5% Triton X‐100 for 10 min to permeabilize cell membrane. The 5% normal goat serum was used for blocking and followed by incubation with MHC I antibody at 4°C overnight and goat anti‐mouse Alexa Fluor 594 at room temperature for 1 h, or MYHC antibody at 4°C overnight and goat anti‐mouse Alexa Fluor 594 or goat anti‐mouse Alexa Fluor 488 secondary antibody at room temperature for 1 h. After wash, the staining images were captured with confocal microscope. For the cross‐sectional areas (CSA) quantification of muscle fiber, for each sample, we selected five random fields in red GAS region and 5 random fields in white GAS region and measured at least 300 fibers in each region using Image‐Pro Plus 6.0 software. For the cell size quantification of adipocyte, we measured five random fields in adipose tissue per mouse using Image‐Pro Plus 6.0 software as previous described (Hu et al., 2021).

### 4.12 ELISA analysis

The mouse Metrnl and IGF ELISA kits were purchased from R&D Systems (DY6679 and MG100). The mouse serum and cultured media were used for measuring METRNL and IGF1 protein contents and the assay was performed as manufacturer's protocol.

### 4.13 RNA sequencing and data analysis

GAS muscles treated with AAV‐GFP or AAV‐NLS‐PGC1α4 were collected and frozen for subsequent RNA extraction. RNA quality was checked using Bio‐analyzer instrument (Agilent, USA), quantified with ND‐2000 (NanoDrop Technologies), and then subjected for cDNA library construction. Briefly, Ploy A mRNA was enriched using Oligo dT magnetic beads from 2 μg total RNA and broken up to 200 bp for each replicate. The double‐strand cDNA was synthesized and purified for end repair, poly A addition and adapter ligation. Then, the products enriched with PCR amplification generated cDNA libraries were subsequently sequenced on Illumina HiSeq™ 3000 platform. The RNA‐seq raw reads generated by Illumina sequencer were performed by trimming of adaptors and removal of low‐quality reads using Skewer (v0.2.2). FastQC (v0.11.5) was used to check the quality of the pretreated data which was mapped to GRCm38 using STAR (2.5.3a). The transcripts were assembled using StringTie (v1.3.1c) and the differential gene transcript expression was analyzed with DESeq2 (v1.16.1). The differential threshold value was *p* < 0.05 and fold‐change>1.5. The KEGG Pathway database (www.genome.jp) was applied for pathway enrichment analysis.

### 4.14 Glucose uptake

For muscle glucose uptake assay, mice were injected locally in GAS muscles with AAV‐GFP or AAV‐NLS‐PGC1α4 for 1 month, and then injected intraperitoneally (i.p.) with the [^14^C]2‐deoxyglucose (DG) (50 μCi/kg, diluted with 0.2 g/kg D‐glucose). The mice were sacrificed after 1 h and GAS muscles were collected quickly. Tissues were weighed and digested in 1 M NaOH (1 mL per 100 mg of tissue, wet weight) at 60°C for 2 h. Extracts were neutralized by addition of 2 M HCl (0.5 mL per 1 mL of 1 M NaOH) and centrifuged 12,000 g for 10 min. Supernatants were mixed with the scintillation cocktail medium Ultima Gold XR (Perkin, US) and performed in Tri‐Carb 4910TR Liquid Scintillation Analyzer (Perkin, US) for glucose uptake analysis. The glucose uptake was normalized to the wet weight of tissue.

### 4.15 Human muscle sample collection and analysis

To determine the PGC1α4 expression levels in human muscle samples, we collected muscle biopsies from the vastus lateralis muscle from 48 human individuals aged between 16 to 88 years old that underwent orthopaedic surgery at Shanghai Jiao Tong University Affiliated Sixth People's Hospital for qPCR and statistical analysis. Human study was approved by Shanghai Jiaotong University Affiliated Sixth People's Hospital. Written informed consent was obtained from all individuals.

### 4.16 Statistical analysis

All data in this study were presented as Mean ± SEM. The normalcy of data was examined by Shapiro–Wilk normality test. Two‐tailed unpaired Student's *t‐*test were used for statistical comparisons between two groups. Two‐way ANOVA with Bonferroni's multiple comparisons was used for comparisons of multiple datasets. ANCOVA analysis was used for comparisons of oxygen consumption, carbon dioxide production and total energy expenditure with body weight as covariant. The correlation between PGC1α4 mRNA levels and human age was analyzed by Pearson correlation analysis. *p* < 0.05 was considered as statistically significant. All statistical analyses were performed using Prism 8 or SPSS software. The *p*‐values were displayed as *p < 0.05, **p < 0.01. ns, non‐significant.

## AUTHOR CONTRIBUTIONS

X.M. and L.X. devised and supervised the project. M.G., J.Z., Y.M. H.Z., J.Y., D.W., X.W. and J.C. performed biochemical and cellular experiments. M.G., J.Z., J.Y., and H.Z. established animal models and C.L., J.Y., Y.M. and Y.Z. participated in animal studies. C.H and Z.Z provided clinical samples. M.G., J.L., S.D., X.M. and L.X. wrote and edited the manuscript.

## CONFLICT OF INTEREST STATEMENT

The authors declare no conflicts of interest.

## Supporting information


**Supplementary Figure S1.** Expression levels of Pgc1α isoforms in muscles during aging.
(A) The mRNA levels of Pgc1α4 in GAS and SOL muscles from 2 months‐old, 18 months‐old and 20 months‐old mice. *n* = 6 per group.
(B) qPCR analysis of Pgc1α1, Pgc1α2 Pgc1α3, in GAS muscles from young (2 months‐old) and aged mice (24 months‐old). *n* = 6 per group.
Data are presented as mean ± SEM and *p < 0.05, **p < 0.01 compared to control group.
Supplementary Figure S2. PGC1α4 locates at cytoplasm in HEK 293 T and NIH‐3 T3 fibroblasts.
(A) Gene sequences comparison between PGC1α1 and PGC1α4.
(B) GFP fluorescence analysis assessing the localization of PGC1α4‐GFP in HEK293T and NIH‐3 T3 fibroblast cell lines transfected with GFP fused PGC1α4. Nuclear was stained by DAPI shown as blue. Scale bar = 100 μm.
(C and D) Immunoblotting analysis of PGC1α4‐GFP cytoplasmic (C) and nuclear (N) levels in HEK293T (C) and NIH‐3 T3 fibroblasts (D) with GFP antibody. Lamin A/C and α‐Tubulin (bottom) were shown as controls for nuclear and cytoplasmic fractions, respectively.
Supplementary Figure S3. NLS‐PGC1α4 locates in nucleus in HEK 293 T and NIH 3 T3 cells.
(A) GFP fluorescence analysis showing the localization of NLS‐PGC1α4‐GFP in HEK 293 T and NIH 3 T3 cells transfected with GFP fused NLS‐PGC1α4. Nuclear was stained by DAPI shown as blue. Scale bar = 100 μm.
(B) Immunoblotting analysis of NLS‐PGC1α4‐GFP cytoplasmic (C) and nuclear (N) levels in HEK293T (B) and NIH‐3 T3 fibroblasts (C) with GFP antibody. Lamin A/C and α‐Tubulin (bottom) were shown as controls for nuclear and cytoplasmic fractions, respectively.
Supplementary Figure S4. NLS‐PGC1α4 induces myoblast viability, proliferation and differentiation.
(A‐D) C2C12 myoblasts treated with Ad‐GFP or Ad‐NLS‐PGC1α4 for 24 h.
(A) CCK8 analysis; (B) The mRNA levels of proliferation related genes such as *Ki67, Cyclin E, Cyclin A2, Cyclin B2* and *Cdk2*; (C) EdU analysis and quantifications; (D) The mRNA expression levels of myotube fusion and differentiation related genes *Myomaker, Myomerger, Caveolin3, MyoD* and *MyoG*;
(E) The protein levels of MYOG and MYHC in myoblasts treated with Ad‐GFP or Ad‐ NLS‐PGC1α4 and differentiated for 96 h;
(F) The representative MYHC immunofluorescence staining and quantification of differentiation index and fusion index. Differentiation index was calculated as the percentage of nuclei in MYHC positive cells to total nuclei. Fusion index was calculated as the ratio of nuclei in MYHC positive cells with more than two nuclei to the total number of nuclei.
Data are presented as mean ± SEM and *p < 0.05, **p < 0.01 compared to control group. Scale bar represents 100 μm.
Supplementary Figure S5. Specificity of NLS‐PGC1α4 delivery in GAS muscle of aged mice.
(A) The schematic diagram illustrated construction strategy of AAV‐GFP or AAV‐NLS‐PGC1α4 locally injected in GAS;
(B‐D) In vivo analysis of specificity of NLS‐PGC1α4 delivery
(B, C) The mRNA levels of Pgc1α4 in GAS, soleus liver, iWAT tissues and heart;
(D) The transduction efficiency showing by the mRNA levels of eGFP in GAS muscle;
(E) Inflammatory gene levels;
(F) average cross section area (CSA) of GAS muscle;
(G) Fiber sizes of slow fibers and all fast fibers revealed by MHC I staining.
Data are presented as mean ± SEM and *p < 0.05, **p < 0.01 compared to control group.
Scale bar represents 100 μm.
Supplementary Figure S6. NLS‐PGC1α4 delivery in GAS muscle exhibited stronger hypertrophic effects than PGC1α4.
(A) The mRNA expression levels of *Pgc1α4, Igf1*, *Igf2, Igfbp2, Igfbp3, Mstn*; (B) The representative H&E staining; (C) The average cross‐sectional areas (CSAs); (D) quantification of muscle fiber size distribution in GAS muscle treated with Lenti‐GFP, Lenti‐PGC1α4 or Lenti‐NLS‐PGC1α4.
Data are presented as mean ± SEM and *p < 0.05, **p < 0.01 compared to control group. Scale bar represents 100 μm.
Supplementary Figure S7. NLS‐PGC1α4 delivery in GAS muscle enhances QU and TA muscle hypertrophy.
(A) Total local motor activity; (B) Food intake; (C) The QU muscle weights; (D) representative H&E staining and quantification of average CSA of muscle fibers in QU muscles; (E) The TA muscle weights; (F) representative H&E staining and quantification of average CSA of muscle fibers in TA muscles of aged mice locally treated with AAV‐GFP or AAV‐NLS‐PGC1α4 in GAS.
Data are presented as mean ± SEM and *p < 0.05, **p < 0.01 compared to control group. Scale bar represents 100 μm.
Supplementary Figure S8. Specificity of PGC1α4 antibody
Immunoblotting analysis of PGC1α4 protein levels with anti‐PGC1α4 antibody.

**Supplementary Table 1.** Serum chemistry tests of Aged mice treated with AAV‐GFP or AAV‐NLS‐PGC1α4 (*n* = 6 per group).

**Supplementary Table S2.** PCR primers used in the study.

## Data Availability

The data that support the findings of this study are available from the corresponding author upon reasonable request.
