# Supplementary material for: AAV‐Mediated nuclear localized PGC1α4 delivery in muscle ameliorates sarcopenia and aging‐associated metabolic dysfunctions
Source: Aging Cell. 2023 Aug 16;22(10):e13961. doi: 10.1111/acel.13961 (PMC10577532; doi:10.1111/acel.13961)

Supplementary Figure S1

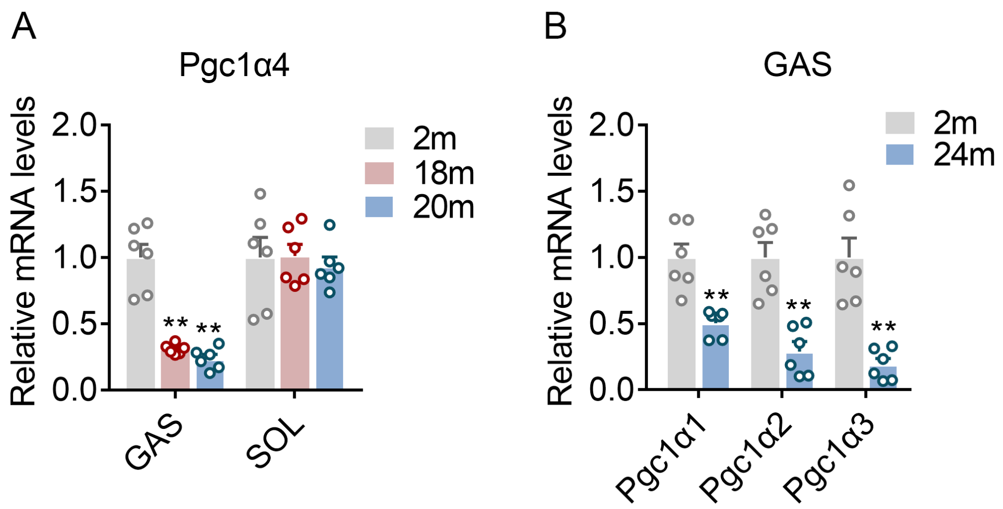

Supplementary Figure S2

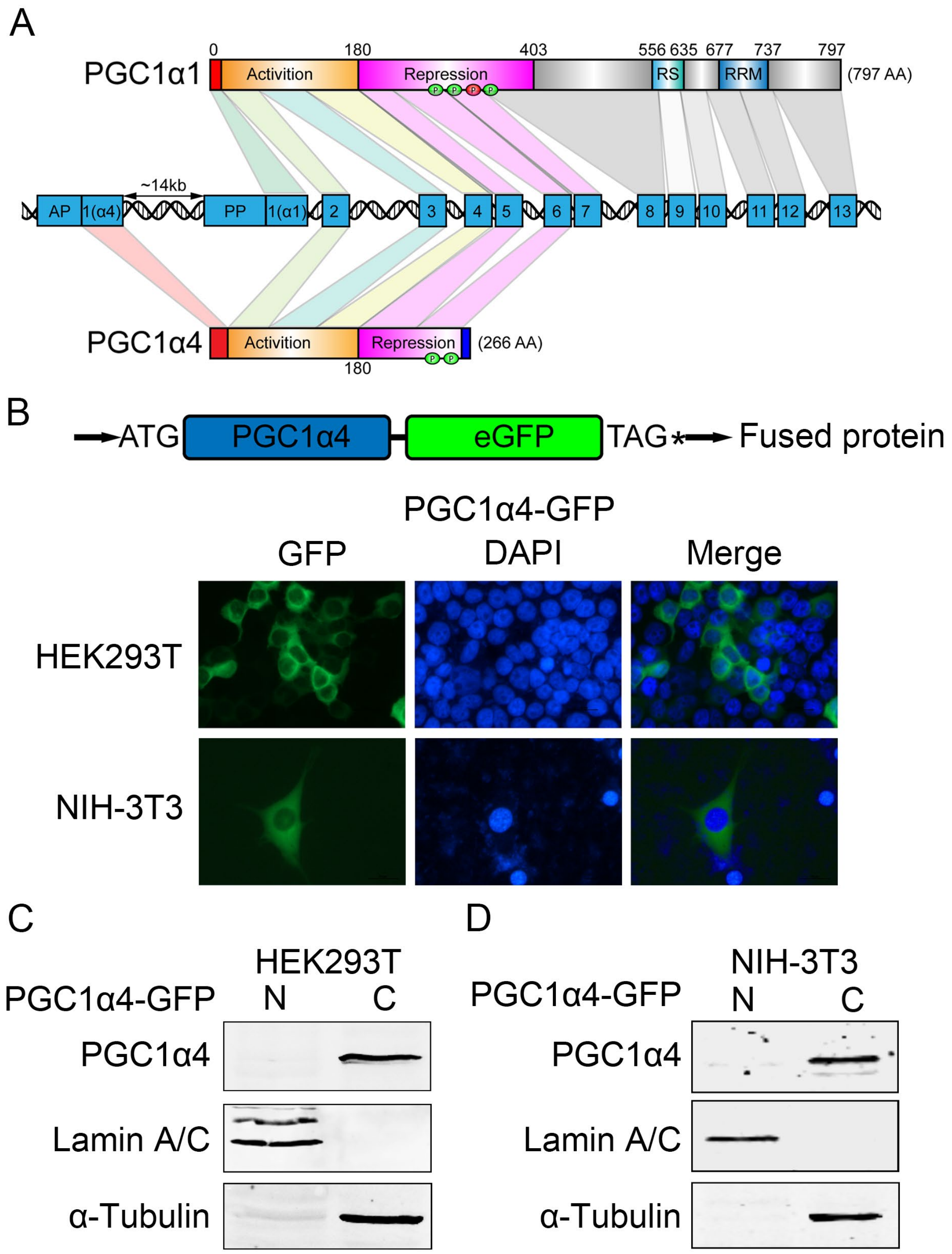

Supplementary Figure S3

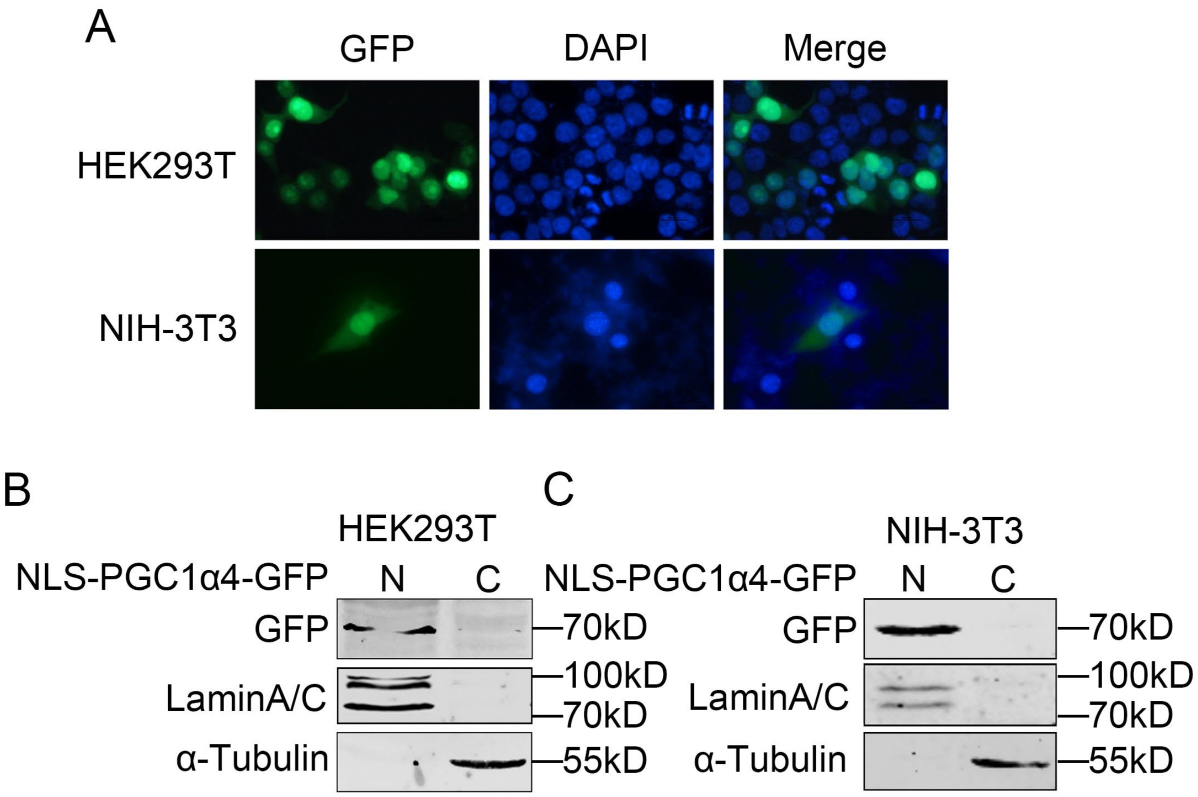

Supplementary Figure S4

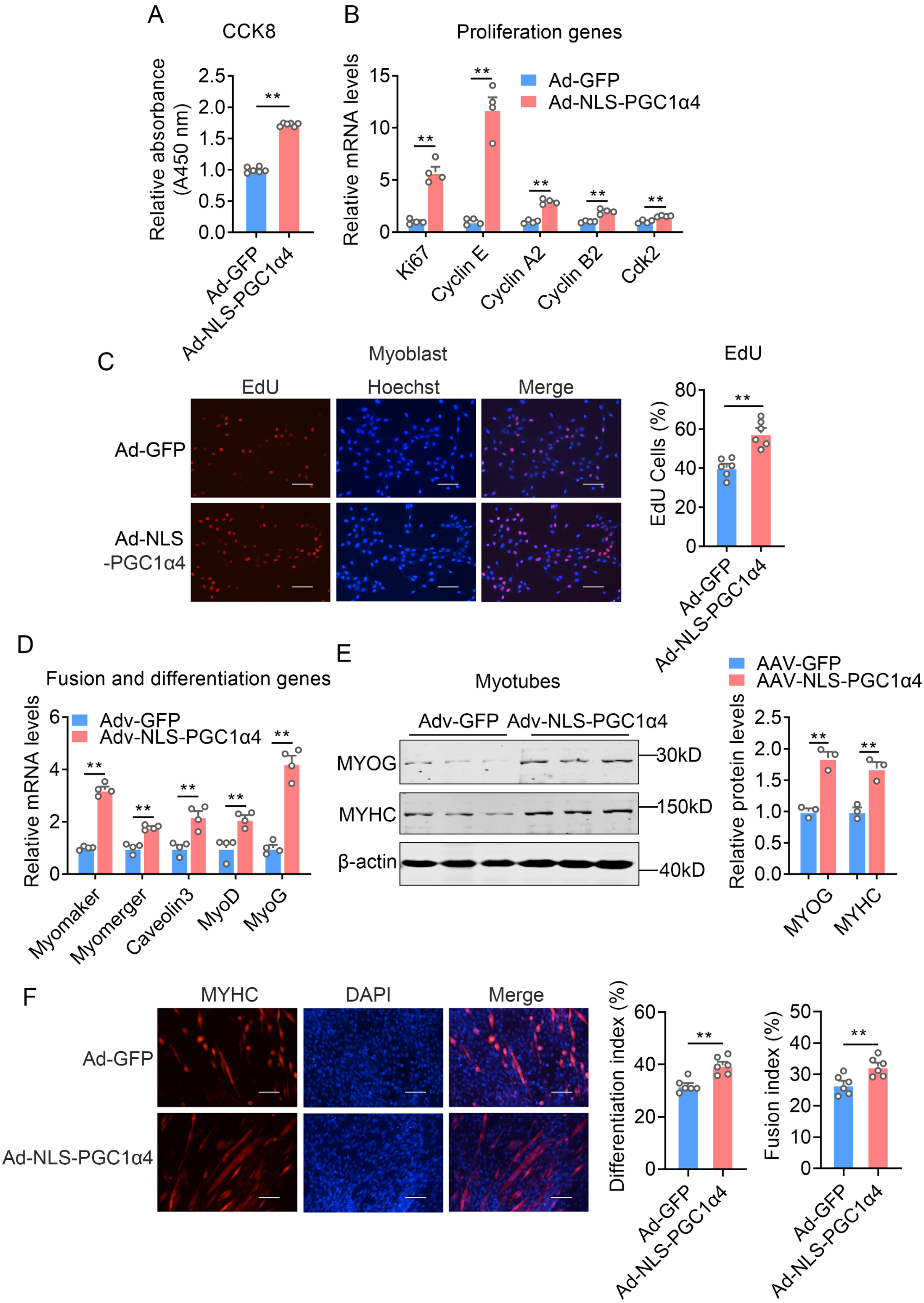

Supplementary Figure S5

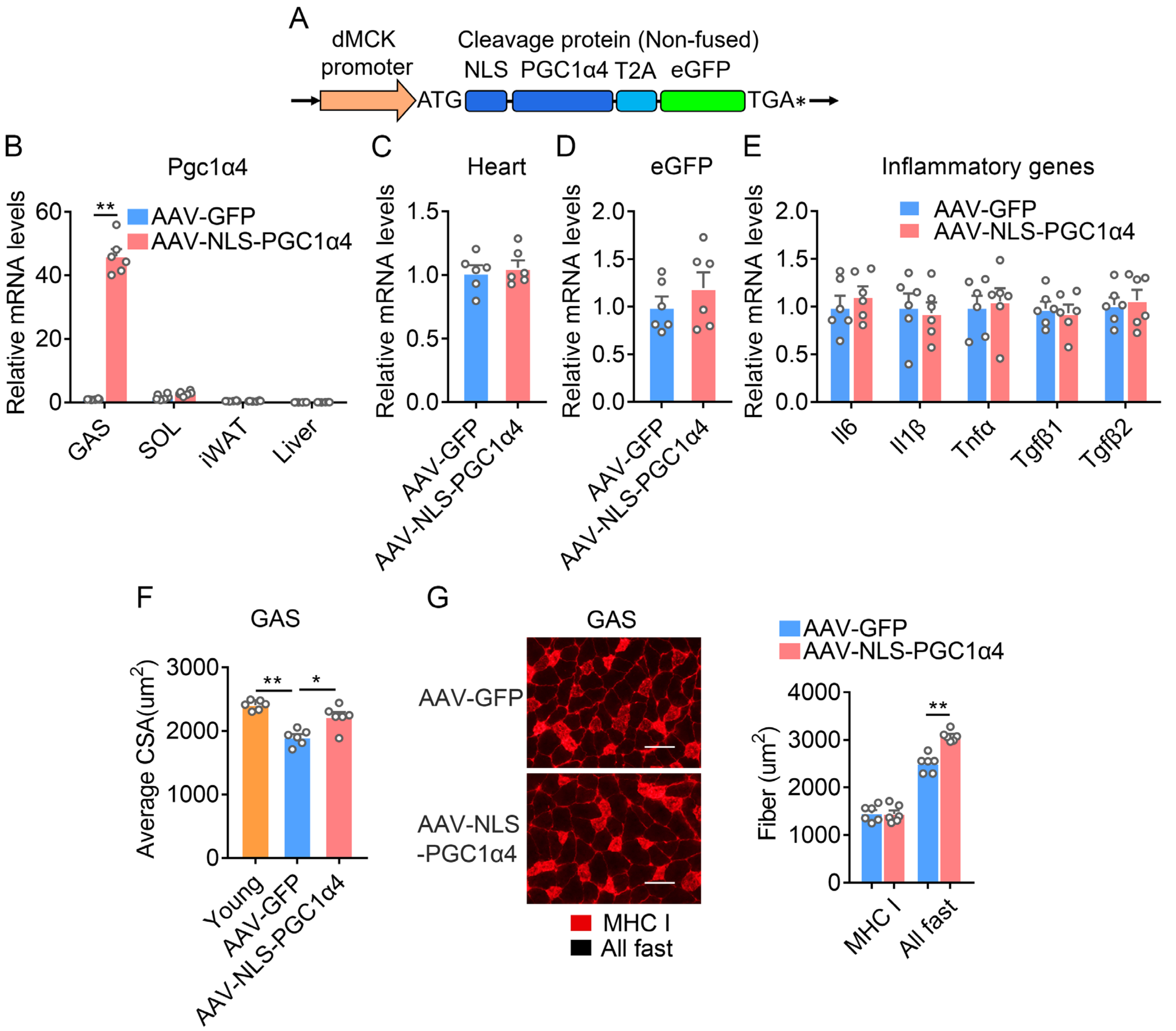

Supplementary Figure S6

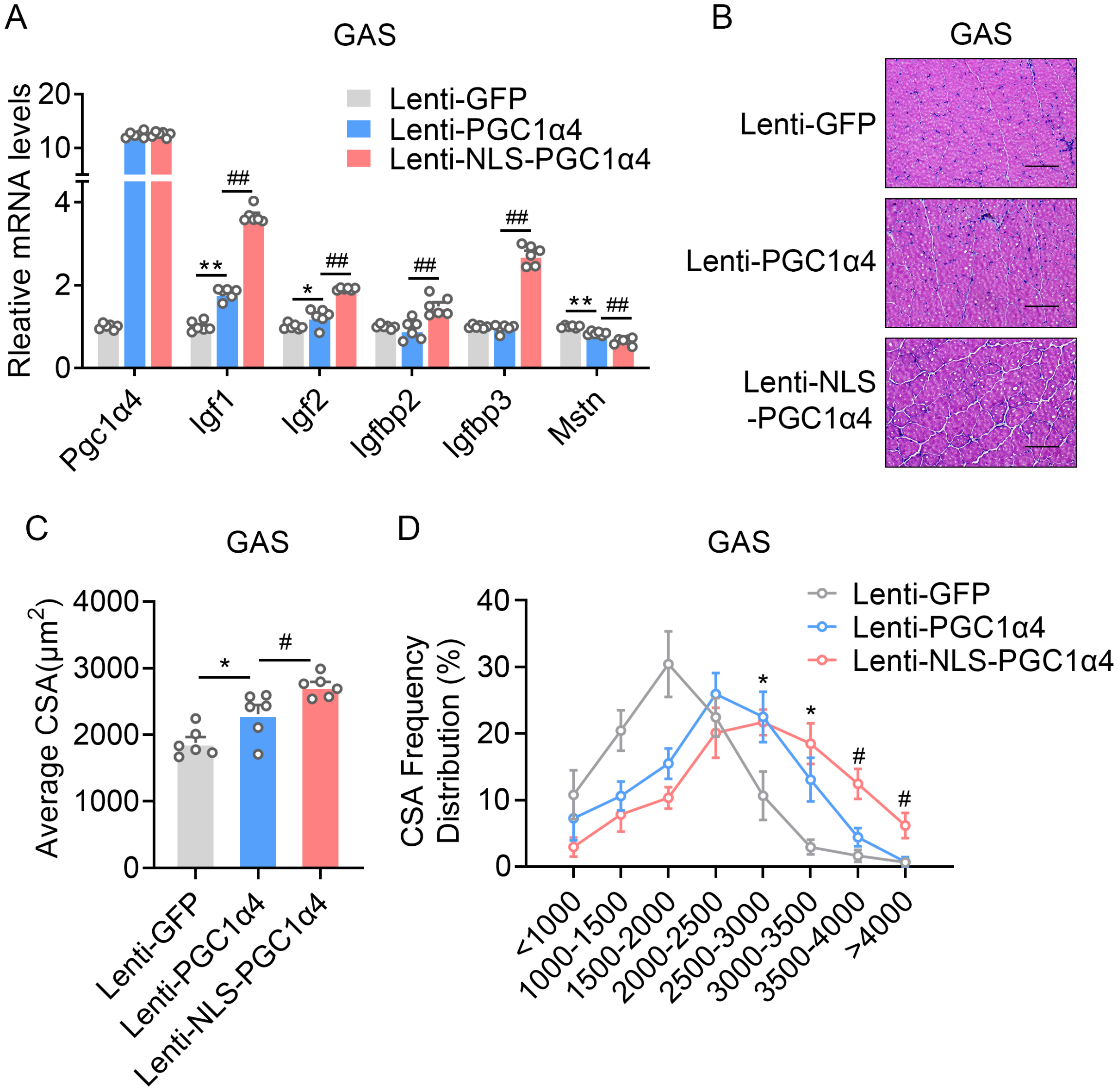

Supplementary Figure S7

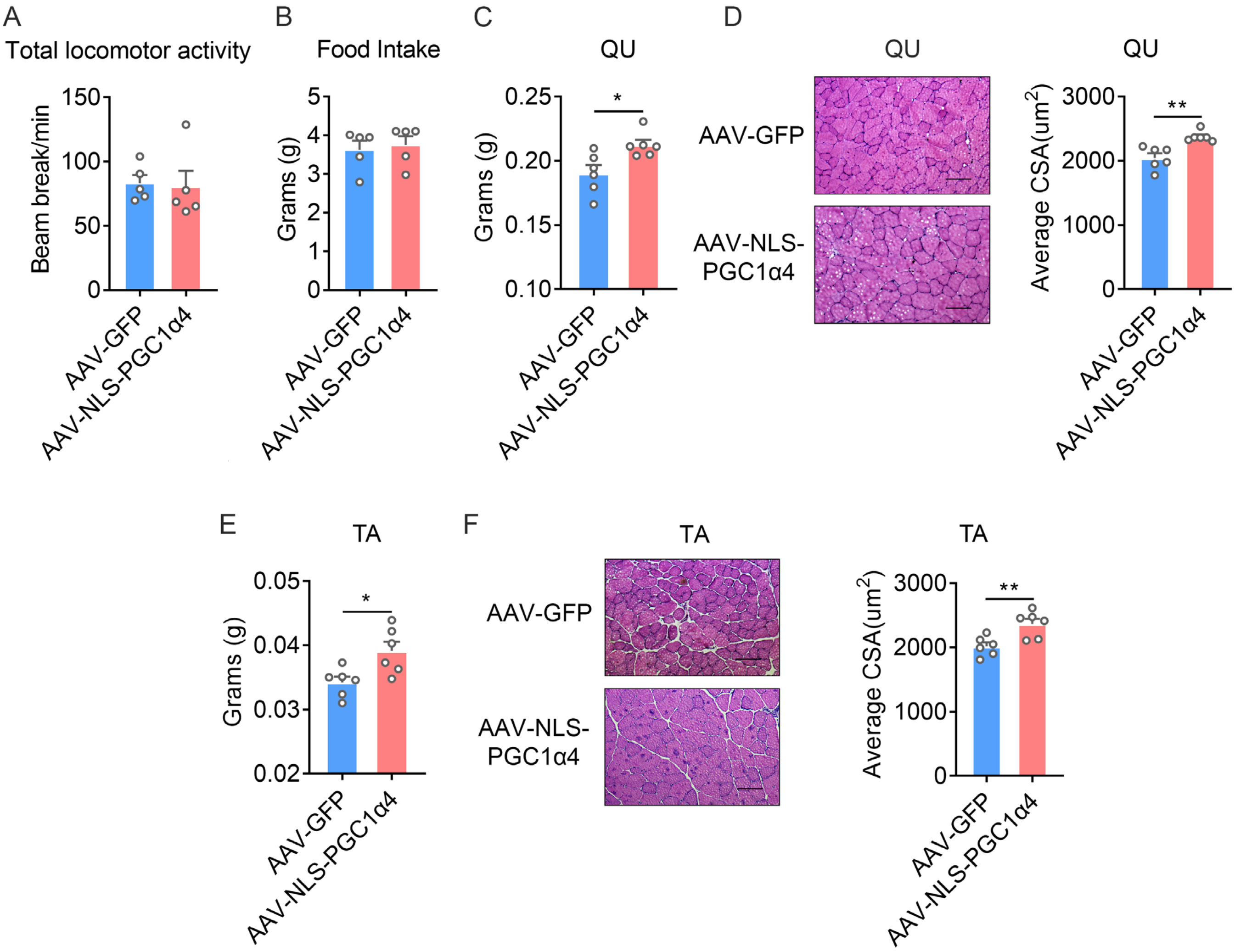

Supplementary Figure S8

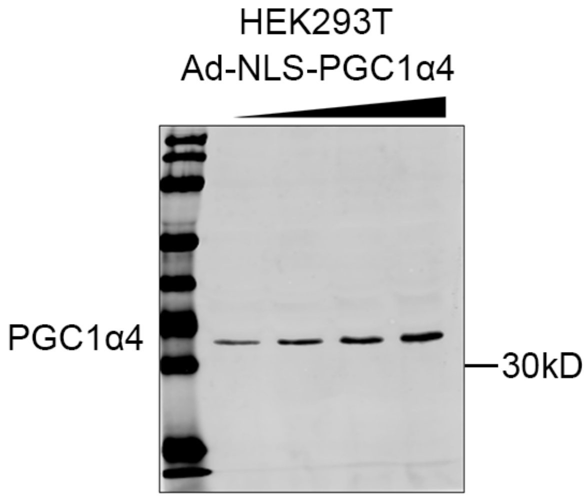

Supplement: Supplementary file 1 — Supplementary Figure S1. Expression levels of Pgc1α isoforms in muscles during aging. (A) The mRNA levels of Pgc1α4 in GAS and SOL muscles from 2 months‐old, 18 months‐old and 20 months‐old mice. n = 6 per group. (B) qPCR analysis of Pgc1α1, Pgc1α2 Pgc1α3, in GAS muscles from young (2 months‐old) and aged mice (24 months‐old). n = 6 per group. Data are presented as mean ± SEM and *p < 0.05, **p < 0.01 compared to control group. Supplementary Figure S2. PGC1α4 locates at cytoplasm in HEK 293 T and NIH‐3 T3 fibroblasts. (A) Gene sequences comparison between PGC1α1 and PGC1α4. (B) GFP fluorescence analysis assessing the localization of PGC1α4‐GFP in HEK293T and NIH‐3 T3 fibroblast cell lines transfected with GFP fused PGC1α4. Nuclear was stained by DAPI shown as blue. Scale bar = 100 μm. (C and D) Immunoblotting analysis of PGC1α4‐GFP cytoplasmic (C) and nuclear (N) levels in HEK293T (C) and NIH‐3 T3 fibroblasts (D) with GFP antibody. Lamin A/C and α‐Tubulin (bottom) were shown as controls for nuclear and cytoplasmic fractions, respectively. Supplementary Figure S3. NLS‐PGC1α4 locates in nucleus in HEK 293 T and NIH 3 T3 cells. (A) GFP fluorescence analysis showing the localization of NLS‐PGC1α4‐GFP in HEK 293 T and NIH 3 T3 cells transfected with GFP fused NLS‐PGC1α4. Nuclear was stained by DAPI shown as blue. Scale bar = 100 μm. (B) Immunoblotting analysis of NLS‐PGC1α4‐GFP cytoplasmic (C) and nuclear (N) levels in HEK293T (B) and NIH‐3 T3 fibroblasts (C) with GFP antibody. Lamin A/C and α‐Tubulin (bottom) were shown as controls for nuclear and cytoplasmic fractions, respectively. Supplementary Figure S4. NLS‐PGC1α4 induces myoblast viability, proliferation and differentiation. (A‐D) C2C12 myoblasts treated with Ad‐GFP or Ad‐NLS‐PGC1α4 for 24 h. (A) CCK8 analysis; (B) The mRNA levels of proliferation related genes such as Ki67, Cyclin E, Cyclin A2, Cyclin B2 and Cdk2; (C) EdU analysis and quantifications; (D) The mRNA expression levels of myotube fusion and different [file ACEL-22-e13961-s003.pdf]
