# Supplementary material for: AAV‐Mediated nuclear localized PGC1α4 delivery in muscle ameliorates sarcopenia and aging‐associated metabolic dysfunctions
Source: Aging Cell. 2023 Aug 16;22(10):e13961. doi: 10.1111/acel.13961 (PMC10577532; doi:10.1111/acel.13961)
Supplement: Supplementary file 2 — Supplementary Table 1. Serum chemistry tests of Aged mice treated with AAV‐GFP or AAV‐NLS‐PGC1α4 (n = 6 per group). [file ACEL-22-e13961-s001.doc]

**Supplementary Table 1. Serum chemistry tests of Aged mice treated with AAV-GFP or AAV-NLS-PGC1α4 (n=6 per group).**

|  | **Tests** | **AAV-GFP** | **AAV-NLS-PGC1α4** | ***P*** |
| --- | --- | --- | --- | --- |
| Renal/proteins | Urea nitrogen (mmol/L) | 10.13±0.66 | 9.85±0.84 | 0.81 |
|  | Uric acid (μmol/L) | 175.3±27.30 | 214.7±24.81 | 0.31 |
|  | Total protein (g/L) | 43.20±1.26 | 42.40±2.07 | 0.76 |
| Enzymes | Aspartate aminotransferase (U/L) | 96.70±13.23 | 77.68±6.59 | 0.21 |
|  | Amylase(U/L) | 698.2±9.00 | 709.2±1.33 | 0.22 |
|  | Creatinine (μmol/L) | 26.62±2.90 | 28.63±4.84 | 0.74 |
|  | Albumin (g/L) | 24.91±0.40 | 24.46±1.18 | 0.75 |
|  | Alanine aminotransferase (U/L) | 40.23±14.76 | 36.37±10.17 | 0.83 |
