# Supplementary material for: AAV‐Mediated nuclear localized PGC1α4 delivery in muscle ameliorates sarcopenia and aging‐associated metabolic dysfunctions
Source: Aging Cell. 2023 Aug 16;22(10):e13961. doi: 10.1111/acel.13961 (PMC10577532; doi:10.1111/acel.13961)
Supplement: Supplementary file 3 — Supplementary Table S2. PCR primers used in the study. [file ACEL-22-e13961-s002.docx]

**Supplementary Table S2. PCR primers used in the study.**

| **Real-time PCR primers used in the study.** | | |  |
| --- | --- | --- | --- |
| Gene name | Primer sequence (5′→3′) | |  |
| mPgc1α4 | Forward | TCACACCAAACCCACAGAAA |  |
|  | Reverse | CTGGAAGATATGGCACAT |  |
| mIgf1 | Forward | CCACACTGACATGCCCAAGA |  |
|  | Reverse | CCTGCACTTCCTCTACTTGTGTTC |  |
| mMstn | Forward | ACGAGTGGATGGTGCGCTGTGTGC |  |
|  | Reverse | TCATTCTGAACGCGCATGAAGCG |  |
| mAtrogin-1 | Forward | CAGCTTCGTGAGCGACCTC |  |
|  | Reverse | GGCAGTCGAGAAGTCCAGTC |  |
| mMuRF-1 | Forward | GTGTGAGGTGCCTACTTGCTC |  |
|  | Reverse | GCTCAGTCTTCTGTCCTTGGA |  |
| mUcp1 | Forward | GGCCCTTGTAAACAACAAAATAC |  |
|  | Reverse | GGCAACAAGAGCTGACAGTAAAT |  |
| mCidea | Forward | TGACATTCATGGGATTGCAGAC |  |
|  | Reverse | CGAGCTGGATGTATGAGGGG |  |
| mPrdm16 | Forward | CCACCAGCGAGGACTTCAC |  |
|  | Reverse | GGAGGACTCTCGTAGCTCGAA |  |
| mFasn | Forward | GCTGGCATTCGTGATGGAGTCGT |  |
|  | Reverse | AGGCCACCAGTGATGATGTAACTC |  |
| mAcc1 | Forward | ATGGGCGGAATGGTCTCTTTC |  |
|  | Reverse | TGGGGACCTTGTCTTCATCAT |  |
| mPparα | Forward | AACATCGAGTGTCGAATATGTGG |  |
|  | Reverse | CCGAATAGTTCGCCGAAAGAA |  |
| mCol1α1 | Forward | CTGGCGGTTCAGGTCCAAT |  |
|  | Reverse | TTCCAGGCAATCCACGAGC |  |
| mCol3α1 | Forward | GGGTTTCCCTGGTCCTAAAG |  |
|  | Reverse | CCTGGTTTCCCATTTTCTCC |  |
| mTgfβ1 | Forward | ATTTGGAGCCTGGACACACA |  |
|  | Reverse | GAGCGCACAATCATGTTGGA |  |
| mActa2 | Forward | CCCAGACATCAGGGAGTAATGG |  |
|  | Reverse | TCTATCGGATACTTCAGCGTCA |  |
| mTnfα | Forward | ATGGCCTCCCTCTCATCAGT |  |
|  | Reverse | TTTGCTACGACGTGGGCTAC |  |
| mIl4 | Forward | GGTCTCAACCCCCAGCTAGT |  |
|  | Reverse | GCCGATGATCTCTCTCAAGTGAT |  |
| mIl13 | Forward | CCTGGCTCTTGCTTGCCTT |  |
|  | Reverse | GGTCTTGTGTGATGTTGCTCA |  |
| mArg1 | Forward | AGACCACAGTCTGGCAGTTG |  |
|  | Reverse | CCACCCAAATGACACATAGG |  |
| mMrc-1 | Forward | TGATTACGAGCAGTGGAAGC |  |
|  | Reverse | GTTCACCGTAAGCCCAATTT |  |
| mClec10a | Forward | CTCTGGAGAGCACAGTGGAG |  |
|  | Reverse | ACTTCCGAGCCGTTGTTCT |  |
| mRetnla | Forward | CCAATCCAGCTAACTATCCCTCC |  |
|  | Reverse | ACCCAGTAGCAGTCATCCCA |  |
| mElovl3 | Forward | TTCTCACGCGGGTTAAAAATGG |  |
|  | Reverse | TCTCGAAGTCATAGGGTTGCAT |  |
| mPgc1α1 | Forward | GGACATGTGCAGCCAAGACTCT |  |
|  | Reverse | CACTTCAATCCACCCAGAAAGCT |  |
| mPgc1α2 | Forward | CCACCAGAATGAGTGACATGGA |  |
|  | Reverse | GTTCAGCAAGATCTGGGCAAA |  |
| mPgc1α3 | Forward | AAGTGAGTAACCGGAGGCATTC |  |
|  | Reverse | TTCAGGAAGATCTGGGCAAAGA |  |
| mKi67 | Forward | GAGGAGAAACGCCAACCAAGAG |  |
|  | Reverse | TTTGTCCTCGGTGGCGTTATCC |  |
| mCyclin E | Forward | GTGGCTCCGACCTTTCAGTC |  |
|  | Reverse | CACAGTCTTGTCAATCTTGGCA |  |
| mCyclin A2 | Forward | GCCTTCACCATTCATGTGGAT |  |
|  | Reverse | TTGCTGCGGGTAAAGAGACAG |  |
| mCyclin B2 | Forward | GCCAAGAGCCATGTGACTATC |  |
|  | Reverse | CAGAGCTGGTACTTTGGTGTTC |  |
| mCdk2 | Forward | TCATGGATGCCTCTGCTCTCAC |  |
|  | Reverse | TGAAGGACACGGTGAGAATGGC |  |
| mMyomaker | Forward | CAGTGAGCATCGCTACCAAGAG |  |
|  | Reverse | GAATGTCACGGCGCATGAAGCA |  |
| mMyomerger | Forward | GTTAGAACTGGTGAGCAGGAG |  |
|  | Reverse | CCATCGGGAGCAATGGAA |  |
| mCaveolin3 | Forward | GATAGACTTGGTGAACCGCGAC |  |
|  | Reverse | ACTTGGAGACGGTGAACGTGGT |  |
| mMyoD | Forward | GCACTACAGTGGCGACTCAGAT |  |
|  | Reverse | TAGTAGGCGGTGTCGTAGCCAT |  |
| mMyoG | Forward | CCATCCAGTACATTGAGCGCCT |  |
|  | Reverse | CTGTGGGAGTTGCATTCACTGG |  |
| eGfp | Forward | CACATGAAGCAGCACGACTT |  |
|  | Reverse | GTCTTGTAGTTGCCGTCGTC |  |
| mIl6 | Forward | TACCACTTCACAAGTCGGAGGC |  |
|  | Reverse | CTGCAAGTGCATCATCGTTGTTC |  |
| mIl1β | Forward | TGGACCTTCCAGGATGAGGACA |  |
|  | Reverse | GTTCATCTCGGAGCCTGTAGTG |  |
| Tgfβ2 | Forward | AAGAAGCGTGCTTTGGATGCGG |  |
|  | Reverse | ATGCTCCAGCACAGAAGTTGGC |  |
| mIgf2 | Forward | CTTCAGTTTGTCTGTTCGGACCG | |
|  | Reverse | GTGGCACAGTATGTCTCCAGGA | |
| mIgfbp2 | Forward | CCTCAAGTCAGGCATGAAGGAG | |
|  | Reverse | TGGTCCAACTCCTGCTGGCAAG | |
| mIgfbp3 | Forward | CCTCAATGTGCTGAGTCCCAGA | |
|  | Reverse | CTTGTCCACACACCAGCAGAAG | |
| m36b4 | Forward | 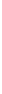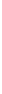AGATTCGGGATATGCTGTTGGC | |
|  | Reverse | TCGGGTCCTAGACCAGTGTTC | |
| mGapdh | Forward | ACAACTTTGGCATTGTGGAA | |
|  | Reverse | GATGCAGGGATGATGTTCTG | |
| hPGC1α4 | Forward | TCACACCAAACCCACAGAGA | |
|  | Reverse | CTGGAAGATATGGCACAT | |
| h18s | Forward | GCAGAATCCACGCCAGTACAAG | |
|  | Reverse | GCTTGTTGTCCAGACCATTGGC | |
| **ChIP qPCR primers used in the study.** | | | |
| pCREB-ChIP | Forward | AGTGCCTCTGTACTAGGGTT | |
|  | Reverse | ACCAGCTACGTTTTAATTCACTGG | |
| β-globin | Forward | AAGCCTGATTCCGTAGAGCCACAC | |
|  | Reverse | CCCACAGGCA AGAGACAGCAGC | |
| **PCR primers for clone constructions in the study.** | | | |
| Pgc1α4 | Forward | GCCACCATGTTGGGATTGTCATCCATGGATTCA | |
| NLS-Pgc1α4 | Forward | AGGGGCTGCCCCCGGGTCACGAATTCGCC  ACCATGCCTAAGAAAAAGAGGAAGGTGCC  TAAGAAAAAGAGGAAGGTGCCTAA | |
|  | Reverse | AGAAGGCTTCCTCTGCCCTCAAGCTTTAAA  AACAAATTTGGTGACTCTGG | |
| CREB | Forward | GCGGATCCGCCACCATGACCATGGAATCTGGAGCCGA | |
|  | Reverse | CCGGAATTCTTACTTGTCATCATCGTCCTTGTAGTCAA  CATCTGATTTGTGGCAGTAAAGGTCC | |
| Mut-CREB (Ser133) | Forward | GAAATTCTTTCAAGGAGGCCTGCATACAG  GAAAATTTTGAATGAC | |
|  | Reverse | GTCATTCAAAATTTTCCTGTATGCAGGCCT  CCTTGAAAGAATTTC | |
| Pgc1α4-promoter (WT) | Forward | CTAGCTAGCCTTGTTGGAGCTAAGAACCTTGA | |
|  | Reverse | GAAGATCTTGGAGAGAATGCCTCCGGTT | |
| Pgc1α4-promoter(Mut-CRE1) | Forward | GAACCACCTGTCTCAACTGCGAGAGCTCC  CTCGAGACTTG | |
|  | Reverse | CAAGTCTCGAGGGAGCTCTCGCAGTTGAG  ACAGGTGGTTC | |
| Pgc1α4-promoter(Mut-CRE2) | Forward | TGACTCCCAGGTGCCCTCCCCTGAGCTCCA  GTGAATTAAA | |
|  | Reverse | TTTAATTCACTGGAGCTCAGGGGAGGGCAC  CTGGGAGTCA | |
